# Capturing the Impact of Patient Portals Based on the Quadruple Aim and Benefits Evaluation Frameworks: Scoping Review

**DOI:** 10.2196/24568

**Published:** 2020-12-08

**Authors:** Melita Avdagovska, Devidas Menon, Tania Stafinski

**Affiliations:** 1 School of Public Health University of Alberta Edmonton, AB Canada

**Keywords:** patient portals, quadruple aim, benefits evaluation framework, mobile phone

## Abstract

**Background:**

Despite extensive and continuing research in the area of patient portals, measuring the impact of patient portals remains a convoluted process.

**Objective:**

This study aims to explore what is known about patient portal evaluations and to provide recommendations for future endeavors. The focus is on mapping the measures used to assess the impact of patient portals on the dimensions of the Quadruple Aim (QA) framework and the Canada Health Infoway’s Benefits Evaluation (BE) framework.

**Methods:**

A scoping review was conducted using the methodological framework of Arksey and O’Malley. Reporting was guided by the PRISMA (Preferred Reporting Items for Systematic reviews and Meta-Analyses) extension for scoping reviews. A systematic and comprehensive search was conducted using the Ovid platform, and the following databases were searched: Ovid MEDLINE (R) ALL (including epub ahead of print, in-process, and other nonindexed citations), EMBASE, and PsycINFO. CINAHL on the EBSCO platform and Web of Science were searched for studies published between March 2015 and June 2020. A systematic gray literature search was conducted using the Google search engine. Extracted data were tabulated based on a coding template developed to categorize the literature into themes and areas of interest.

**Results:**

A total of 96 studies were included for data extraction. The studies were categorized based on the QA dimensions, with strict adherence to the definitions for each dimension. From the patients’ perspective, it was determined that most evaluations focused on benefits and barriers to access, access to test results, medication adherence, condition management, medical notes, and secure messaging. From the population perspective, the evaluations focused on the increase in population outreach, decrease in disparities related to access to care services, and improvement in quality of care. From the health care workforce perspective, the evaluations focused on the impact of patients accessing medical records, impact on workflow, impact of bidirectional secure messaging, and virtual care. From the health system perspective, the evaluations focused on decreases in no-show appointments, impact on office visits and telephone calls, impact on admission and readmission rates and emergency department visits, and impact on health care use. Overall, 77 peer-reviewed studies were mapped on the expanded version of the BE framework. The mapping was performed using subdimensions to create a more precise representation of the areas that are currently explored when studying patient portals. Most of the studies evaluated more than one subdimension.

**Conclusions:**

The QA and BE frameworks provide guidance in identifying gaps in the current literature by providing a way to show how an impact was assessed. This study highlights the need to appropriately plan how the impact will be assessed and how the findings will be translated into effective adaptations.

## Introduction

### Background

Electronic patient portals are secure websites tied to an institutional electronic health record (EHR) system from which patients can view their medical information. These types of records are populated with a person’s lifetime health history. The information comes from various sources, including community clinics, hospitals, physicians, pharmacies, and laboratories. Many allow patients to view appointments, medical test results, and medication therapies and communicate with their health care providers through a secure platform [1,2]. Although evidence suggests that the implementation of patient portals can have a positive impact on patient care and patient outcomes, many health systems have been slow to adopt them because of various concerns [3-6].

Patients have expressed concerns about accessing and maintaining health information in a private and secure manner [2,7,8]. As these portals are web-based tools, some worry that their data could be seen by other individuals and by insurance companies [9]. Others have expressed concerns around optimal design and functionality [2]. Furthermore, not all patients have access to a computer, smartphone, or tablet to access their record [10].

Health care providers have conveyed concerns about the implementation, availability, and impact of portals on the patient-provider relationship. Physicians continue to be concerned that portals will increase their workload, without a mechanism for remuneration in fee-for-service models [11]. In addition, there are some uncertainties around physician obligations with respect to portal use [9,11-14]. Providers are concerned that this type of health technology will start replacing office visits and thus have an impact on the way care is provided [15]. Another practice-related concern is the absence of transparency surrounding the provider’s role and accountability with respect to patient portals and protection of patient data [3].

For governments, the challenge of implementing portals has been the upfront cost of establishing an effective and efficient system [14,16,17]. Furthermore, studies show that the majority of health information technology investments are struggling to achieve the anticipated benefits [18-21].

Despite continuing extensive research in this area, the implementation and adoption of these systems remains a convoluted process. First, although various reviews [22-31] have been conducted in this area recently, none have focused on the impact of patient portals within the context of the 4 specific dimensions of the Quadruple Aim (QA) framework [32]. Second, the majority of published reviews have examined one characteristic, such as engagement, barriers and facilitators, outcomes, or communication, and these reviews usually emphasize patients or health care providers. Third, no reviews have looked at which patient portal functions are most commonly used to evaluate impact based on the Canada Health Infoway’s Benefits Evaluation (BE) framework [33].

Two frameworks for analysis were incorporated: the QA and BE frameworks. The QA framework is a modification of the established Triple Aim Framework [34] of health care improvement, which focuses on evaluating 3 dimensions of care: improving the health of populations, improving the patient and caregiver experience, and reducing the per capita cost of health care. The QA framework [32] focuses on improving the work life of providers as the fourth dimension, providing a more comprehensive approach to the evaluation of health technology. Definitions for each of the QA dimensions were used to identify measures as they related to what is considered significant to the patient (ie, preferences, satisfaction, communication, access, engagement, use, etc), population (ie, equity, access, disparities, etc), health system (ie, costs, utilization, etc), and health workforce (ie, satisfaction, workload, preferences, etc).

The BE framework [33] as described and expanded by Lau et al [35] was used to organize measures from peer-reviewed studies. The BE framework was introduced in 2006 by Canada Health Infoway to determine how evaluations might be conducted to capture and measure relevant indicators. The indicators are divided into 8 categories (system quality, information quality, service quality, quality, access, productivity, use, and use satisfaction) and 20 subcategories. Lau et al [35], in their review of systematic reviews, added patient and provider, implementation, and change/improvement as additional categories, which were incorporated in this study.

### Objectives

The purpose of this study is to explore what is known about patient portal evaluations and provide recommendations for future endeavors. It specifically addresses the following research questions:

1. How is the impact of patient portals measured from the standpoint of the 4 specific dimensions (patients, population, health care workforce, and health system) of the QA framework?
2. What components from the BE framework (as expanded by Lau et al [35]) are most commonly evaluated to measure impact?

## Methods

### Methodology

A scoping review was conducted following the 5 steps identified by Arksey and O’Malley [36]: identifying the relevant research question; identifying the various relevant studies in this field; selecting studies; charting the data; and collating, summarizing, and reporting the results. This type of review is recognized as particularly useful for exploring topics with inconsistencies in the current evidence, as it appropriately captures broad and ambiguous topics and approaches.

In this study, *impact* is defined as the *overall effects, direct or indirect, of a policy, strategy, program or project* (in this case, patient portals) [37].

No ethics approval was sought or required for this study, as it did not involve any human subject because it was only focused on reviewing the literature.

### Data Sources and Searches

To capture the wide array of studies that may be relevant to this topic, all study designs were included. A gray literature search was developed to capture all relevant publications, such as government and evaluation reports. Publications that study the same intervention in the same set of patients were matched and classified as a single study.

The inclusion and the exclusion criteria are described in Textbox 1.

**Inclusion and exclusion criteria.**

Inclusion criteria

- Studies with any defined impact and outcomes of tethered patient portals or personal health records
- Studies with relevant impact and outcomes of tethered patient portals or personal health records

Exclusion criteria

- Studies without any defined impact and outcomes of tethered patient portals or personal health records
- Studies with no relevant impact and outcomes of tethered patient portals or personal health records
- Studies describing impact and outcomes of untethered patient portals or personal health records
- Non-English language
- Documents published before 2015
- Abstracts
- Commentaries
- Opinions
- Articles summarizing study findings
- Clinical trials and clinical trial recruitment

With support from an experienced medical information specialist, a search strategy for peer-reviewed papers was developed and tested through an iterative process. Another senior information specialist peer reviewed the strategies before execution using the peer review of electronic search strategies (PRESS) checklist [38]. The following databases were searched using the OVID platform: Ovid MEDLINE, including epub ahead of print, in-process, and other nonindexed citations, EMBASE, and PsycINFO. CINAHL (Cumulative Index of Nursing and Allied Health Literature) on the EBSCO platform and Web of Science were also searched. All searches were performed on June 8, 2020. Strategies used a combination of controlled vocabulary (eg, *Patient Portals*, *Electronic Health Records*, *Patient Access to Records*) and keywords (eg, *health portal*, *EHR portal*, *ehealth patient access*). Vocabulary and syntax were adjusted across databases. Specific details regarding the strategies appear in Multimedia Appendix 1. After removal of all duplicates, the total number of articles remaining was 34,128. Citations retrieved via the searches of electronic databases were imported to Covidence, a Cochrane-supported software designed for conducting reviews.

A systematic gray literature search was conducted using the Google search engine in Edmonton, Alberta, Canada, between February 13 and 25, 2020. The search term *patient portal* was combined with the terms *impact* or *outcome*. The first 100 hits were considered from each combination. In addition, organizational websites of Canada Health Infoway, Canadian Agency for Drugs and Technologies in Health, the National Institute for Health and Care Excellence, the International Network of Agencies for Health Technology Assessment, and the World Health Organization were scanned. Finally, the reference lists of the included articles were searched manually.

### Study Selection

The relevance of the retrieved studies was assessed using the inclusion criteria to ensure that they were related to the topic of this study. All citations were reviewed by titles and abstracts. All articles that focused on topics other than patient portals or personal health records were eliminated. Thus, 2259 articles remained, the titles and abstracts of which were screened independently by 3 researchers (MA, TS, and DM) who applied the inclusion and exclusion criteria. For quality assurance, 9.96% (225/2259) of the articles were reviewed by more than one researcher. No significant discrepancies were noted.

Potentially relevant citations were then retrieved and divided among the 3 researchers for screening using the same inclusion and exclusion criteria. For quality assurance, 10% (9/96) of the papers were reviewed by more than one researcher. No significant discrepancies were noted.

Studies that focused on *untethered* patient portals or personal health records, which were not available in English or were conference abstracts, unpublished dissertations, opinions, or editorials, were excluded.

A total of 10 reviews [22-31] that fit the inclusion criteria were identified. References from each of the reviews were scanned, and 58 articles that met the inclusion criteria were identified. Of these, 7 were reviewed and determined to be captured in the studies already included.

### Data Extraction

As is customary in scoping reviews, an iterative approach was used to extract data from the selected studies. A data extraction form was developed and reviewed to categorize the literature into themes and areas of interest, which varied by study type. The following elements were considered, discussed, and incorporated in the data extraction form (tabular format): authors, title, publication date, country, type of source, study setting, research questions, aims, data collection methods, vendor, patient portal name, patient portal functions, deployment date, end date of the project, type of evaluation, number of patients impacted, number of staff impacted, intervention, control, length of follow-up, benefit area, net benefit indicators, measures, definition of measures, tools used to measure, results and key themes identified in the study, identified success factors, identified challenge factors, identified recommendations, and other considerations, thoughts, and notes.

The extraction form was piloted with several sources and any identified issues were corrected.

### Quality Assessment

As scoping reviews include a broad range of information sources and topics, no critical appraisal of the quality of the included papers was conducted. Although there are various suggested [39] approaches for accomplishing this, there is no consensus among experts in the field on this matter.

### Data Analysis and Synthesis

Extracted data were tabulated based on a coding template developed to categorize the literature into themes and areas of interest, which varied by study type and QA perspective. The studies were categorized based on the QA dimensions, with strict adherence to the definitions for each dimension [32,34]. Furthermore, the evaluated patient portal functions were mapped onto the BE framework [33,35]. A descriptive, analytical approach was used to summarize the outcomes of the studies. In addition, a list of the various terms/outcomes/variables that were used to describe *impact* was compiled.

## Results

### Results of the Literature Search

**Figure 1:**
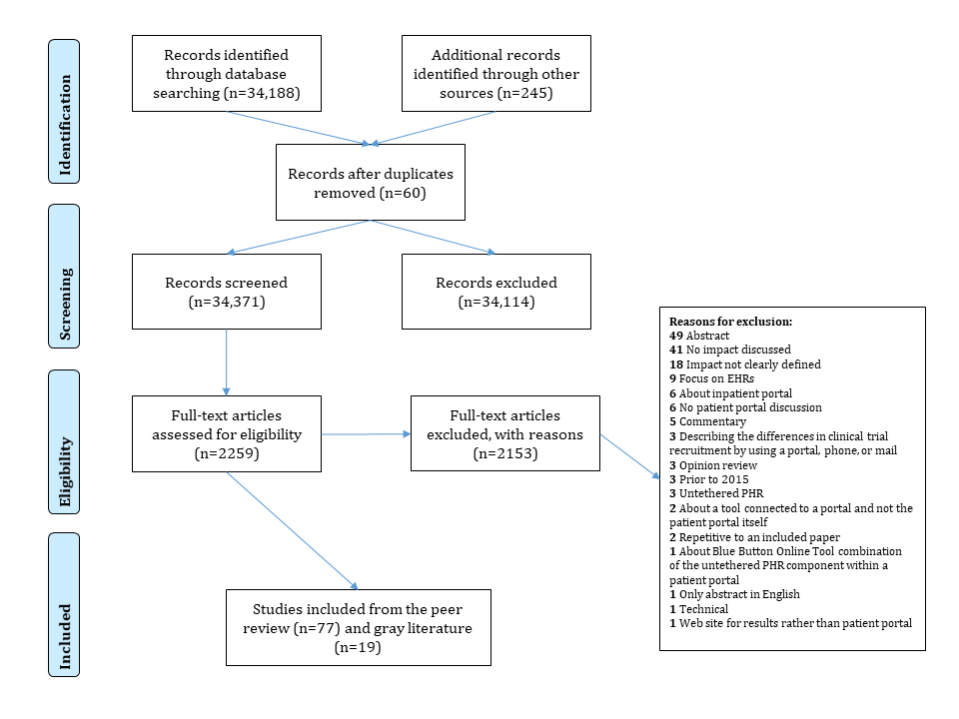
PRISMA (Preferred Reporting Items for Systematic reviews and Meta-Analyses) flow diagram. EHR: electronic health record; PHR: personal health record.

A total of 34,371 citations were identified through the peer-reviewed and gray literature searches. From these, 241 citations were considered for a full-text review. In total, 96 studies were included for data extraction. The search strategy results are described using the PRISMA (Preferred Reporting Items for Systematic reviews and Meta-Analyses) flow diagram, as shown in Figure 1.

### Overall Description of Included Studies

The 96 included studies employed a variety of methods (mixed, n=21; prospective, n=8; qualitative, n=12; randomized controlled trial [RCT], n=3; retrospective, n=25; and survey, n=27). They were conducted between 2015 and 2020 (2015, n=21; 2016, n=25; 2017, n=14; 2018, n=15; 2019, n=13; and 2020, n=8) in Australia (n=2), Canada (n=21), China (n=1), Finland (n=1), the Netherlands (n=2), Norway (n=1), Spain (n=1), Sweden (n=6), the United Kingdom (n=1), and the United States (n=60). Although not all studies specified a clinical problem, most were related to cardiology procedures and conditions, depression, posttraumatic stress disorder, HIV, substance use disorder, anxiety, schizophrenia, neurological issues, pregnancy, and diabetes. A summary of the included studies is captured in Multimedia Appendix 2 [27,40-136].

Overall, 32 peer-reviewed studies [40-71] evaluated the patient portal in general, with all available functions. Secure messaging and medical notes (OpenNotes) were the most commonly evaluated individual portal functions (11 studies each). Five studies assessed multiple functions, such as secure messaging and refills or secure messaging and medication reminders. Gray literature studies evaluated the patient portal, in general, as their focus was on appraising various identified net benefit areas (ie, quality, access, system use, etc), and patient and provider satisfaction with the available functions.

### QA Dimensions

The following sections summarize the studies according to the QA dimensions (Multimedia Appendices 3-6) [27,43,44,46-48, 53,55-58,60-63,65,67,68,72-103,105-114,119,126,127,129].

#### Patient Perspective

The patient perspective was explored in 44 peer-reviewed [43,44,46-48,53,55,56,58,60-63,65,67,68,72-99] and 15 gray literature [100-114] studies. Several methods (mixed, n=12; observational, n=5; qualitative, n=5; RCT, n=1; retrospective, n=14; and survey, n=22) were applied to gain insights from patients through surveys, interviews, focus groups, and administrative data. Of the 59 studies, 35 were from the United States, 17 from Canada, 2 from the Netherlands, and 1 each from Australia, China, Norway, Sweden, and the United Kingdom. The studies usually explored the impact of the patient portal in general, with only a few focusing on various portal functions, such as test results, medical notes (OpenNotes), secure messaging, or prescription refills.

##### Benefits of Access

Many of the studies [47,68,77,80,82,100,101,109-112,114] have explored patient experiences with access to patient portals and subsequently access to their own medical information. All of the studies reported that users were highly satisfied with the access, and in one study, 97% of survey respondents stated that they would definitely or probably recommend the portal to other clients and families [110].

Moll et al [58] observed that patients considered access to information as a means of patient empowerment and involvement. This was also observed by Crouch et al [43], who found that the use of the portal was associated with significantly higher levels of patient activation and levels of patient satisfaction around timely appointments, care, and information. In addition, studies found that if the health care provider encouraged access, the likelihood of patient enrollment and use of the service was much greater [47,62,73].

Reed et al [60] found that 9 of 10 patients believed the portal improved their health care convenience. In addition, access to the information allowed for better engagement of patients with providers, as they had more knowledge about their health [61]. Furthermore, the information eliminated the time pressure felt during short appointments [60]. A different study reported that 28% of patients/families avoided making a telephone call to a health care provider because they could access health information electronically [111]. Convenience was also noted in the findings of another study, in which 27.2% of patients reported savings in terms of time to travel, time off work, gas, and parking [63]. In a study by Graham et al [48], 48% of users reported avoiding a clinic visit and 2.7% avoided an emergency department visit. Convenience was also described because of the ability to make web-based appointments rather than by calling the office [106]. In another study, 27.4% of patients indicated that they had used the patient portal at least once to request an appointment with a primary care provider rather than making a telephone call [114].

Access to patient portal information decreased stress levels because of appointment preparedness [114]. One study reported that 40% of respondents found the portal useful, as it allowed them to plan and follow up on upcoming appointments [67]. In another study, 60% of respondents felt the portal had resulted in an increased sense of partnership with their health care provider, compared with 50% of respondents who felt the portal had positively impacted their relationship with their health care provider [109].

Although most of the studies reported a positive impact because of portal access, one study found little evidence that the portal led to feelings of greater involvement in the care process, improved ability to express concerns to providers or enhanced relationships with providers, or reduced number of in-person visits [55].

##### Barriers to Access

Several studies described patient-reported barriers to access to patient portals [67,73,108]. These barriers were related to privacy, security, and technical difficulties when patients attempted to enroll or use the patient portal. Giardina et al [73] found that 52.6% of the participants wanted portal improvements in terms of display, usability, and notifications. Approximately 24% of patients had higher expectations based on their idea of what functionalities a patient portal should provide, whereas 22% experienced usability problems [67]. Another study [108] found that low user adoption was because of technical issues experienced by patients during enrollment. Patients were unwilling to spend extra time to find solutions and eventually abandoned the creation of an account [108].

##### Access to Test Results

The most commonly used portal function was the access to laboratory or diagnostic test results. The studies assessing this function concluded that the impact was multifaceted, providing patients with convenience, knowledge, tracking of information, decreased anxiety, and the need for fewer appointments [58,61,68,73,77,83,97,103,106,113]. Visual indications were used to determine whether the test results were normal or abnormal (ie, green or red color). Patients described laboratory results as the most important information for them to access. Getting real-time information of laboratory tests before appointments led to increased awareness about personal health. A study found that the availability of web-based radiology reports was associated with increased patient use of the system, with a likelihood ratio of 2.63 [97]. The rates of laboratory test–related anxiety were low. Another study found that 68.41% wanted access to new information on the same day or after a day, whereas the remaining patients were willing to wait anytime between 2 weeks and 1 month, depending on the type of test [58].

Although access to test results was described as the most appreciated function, several studies found that it led to concerns. Two studies [73,77] addressed the concern of the inability of patients to completely understand the laboratory or diagnostic test results in their medical records. Both studies found that patients did not feel that health care providers gave sufficient information when commenting on results. The study by Giardina et al [73] showed that 63.2% of the participants reported that their physician did not include a note explaining the result. Most often, the medical terminology used to describe the test results led to the inability to interpret if they were normal or abnormal. The problem of not understanding results led to apprehension and anxiety until the patient was able to connect with their provider and obtain clarification [73,77]. A study found that one of every 6 patients who underwent magnetic resonance imaging or computerized tomography scans reported a clear understanding of their results when first receiving them through the portal [83]. Patients wanted to receive all their results, even the abnormal ones, but they needed more timely notifications and guidance by their provider in interpreting them [90]. However, some patients preferred to have the potentially concerning test results verbally communicated by a health care professional [90].

##### Medication Adherence

Several studies have explored the correlation between patient portal use and medication adherence because of web-based reminders for refills and requests for prescription renewals [44,81,85,86,89,94,106,109,112]. A study found that once new users were given mobile access to the portal, there was a statistically significant improvement in adherence to oral diabetes drugs and lower glycemic levels [81]. These improvements were greater among patients with a higher clinical need at baseline (glycated hemoglobin [HbA_1c_] level >8%) and more modest but still statistically significantly better among patients with lower initial glycemic levels [81]. Wright et al [86] found that adherence to antihypertensive medications increased if patients had access to their progress notes. The secure messaging function had a similar effect on the likelihood of achieving HbA_1c_ control, as patients who only read email also had significantly lower mean HbA_1c_ values than that of nonusers [44]. Similarly, another study observed that secure messages had the greatest impact on diabetes medical management considerations in terms of HbA_1c_ test completed or missed therapy intervention [96].

One study found small, statistically significant, meaningful improvements in physiological measures among patients with diabetes who initiated and sustained the use of refills through the patient portal [94]. The refill function, in combination with secure messaging, had a greater impact on HbA_1c_ levels. Another study observed stable refill adherence over time among portal users compared with small declines among nonusers [85]. Satisfaction with the refill portal function was high, as 69% would recommend e-refill requests to other patients, family, or friends, and 63% would request all or most of their prescription refills electronically [112]. Furthermore, a nation-wide survey in Canada found that when prescriptions were lost or damaged, 17% of patients decided to go without the medication [106]. Consequently, portals were determined to be effective as a tool to update medication lists and had the potential to augment the existing phone-based medication update process [89].

##### Condition Management

Patients described portal access as a way to monitor their conditions and be more proactive in their care. The severity of the disease predisposed the level of use [62]. A study that measured the acceptability and clinical outcomes of the portal in parents of children with moderate or severe asthma observed that parents used the portal as a decision-support tool that allowed for improved knowledge about the condition [46]. The more severe the child’s condition, the higher the acceptance and use of the portal [46]. Crouch et al [43] concluded that higher portal use was associated with positive clinical and behavioral characteristics related to the management of chronic conditions. A study found that access to the portal added value in the received care during pregnancies [47]. Broman et al [87] found that portal use was effective in postoperative care and follow-up. Another study reported that 88% of survey respondents reported that portal access allowed for better health management [109].

However, a few studies found that portal use did not enhance patients’ experiences. Two-thirds of persistent users responded that they did not feel that the portal supports them in most lifestyle choices [62]. A study observed that portal use among patients with chronic conditions enrolled in a care coordination program did not demonstrate a statistically significant improvement in self-efficacy and perception of health status [65].

##### Medical Notes

Access to medical notes (usually referred to as OpenNotes in the literature) through patient portals was another component of several studies. A study found that almost all patients described enhanced comprehension about their disease and care because of access to clinicians’ notes, as the notes refreshed their memory and clarified their understanding of visits [74]. Patients reported that the medical notes eased their uncertainty, relieved anxiety, and facilitated control [74]. Denneson et al [75] found that reading OpenNotes helped 49% of patients have feelings ranging from very to extremely in control of their health care. Another study observed that access to notes increased patient trust toward their health care providers [92]. Notes not only provided a way for patients to learn about their condition but also checked for any inaccuracies and made face-to-face time more effective [72].

Higher levels of reading notes were associated with higher shared decision-making levels [78]. A study observed that patients who read >4 notes were 15% more likely to have high scores for clinician effort in helping them understand health issues and 16% more likely for clinician efforts to include them in the plan of care [78]. The study concluded that there was a strong correlation between shared decision making and the transparency provided by OpenNotes. A similar finding was observed by Walker et al [79], who found that transparency through notes helped patients feel more engaged in their care.

Caregivers found access to clinicians’ notes valuable. A study found that 55% of caregivers reported reading notes helped them remember to get the patient’s tests done, and 92.3% reported reading notes helped them understand the reason for the patient’s referral to a specialist [76]. The same study found that caregiver access to notes had little to no negative impact on caregiver-provider relationships [76]. Wolff et al [98] found that 35.5% of caregivers viewed doctor notes because they were unable to visit.

For subsets of patients, access to medical notes increased their anxiety levels [74]. One study found that 26% of the patents experienced stress or worry sometimes, whereas 8% reported often or always [75]. The study also reported that 18% of patients felt upset sometimes after reading their notes, compared with 8% who reported often or always. Furthermore, race and ethnicity affected the levels of access to the notes. Minorities and patients with a lower socioeconomic status accessed notes at lower rates than patients who were White and had a high socioeconomic status [93].

##### Secure Messaging

The secure messaging function was most commonly used to request clarification, ask condition-related questions, or inform providers or patients about any health changes [99]. Secure messages were described as a tool to recognize and decrease any gaps in care [96]. A study found that secure messaging allowed for efficient bidirectional radiologist-patient communication [97]. Haun et al [88] noted that the majority of the respondents used secure messaging at least once a year, and less than 15% reported never using secure messaging. The same study observed that patients were satisfied with secure messaging, as it provided a safe and secure communication tool that was easy to use and saved time [88]. Another study found that patients reliably read messages sent by their physicians, and the rate of unread messages was 3.1% at 21 days [84]. Furthermore, secure messaging improved the management of clinical outcomes. Petullo et al [95] found that active secure messaging use was associated with a 0.156% lower HbA_1c_ compared with inactive patients (*P*<.001) and a 0.263% lower HbA_1c_ compared with active nonusers (*P*<.001). Similar rates were observed by Devkota et al [44], in which patients who read and wrote emails had significantly (*P*<.001) lower average HbA_1c_ values compared with nonusers. A study observed that patients who used the portal, compared with nonusers, were 24% more likely to achieve blood pressure control; however, after adjusting for sociodemographic factors, this association was no longer present because of low rates of portal use among minorities and disadvantaged patients [56].

The main barrier to the use of secure messaging was the unresponsiveness of health care providers to the messages sent by patients, which led to increased rates of telephone calls [67].

#### Population Perspective

Enhancing population health through decreasing disparities and elevating access to needed health services was explored in 5 peer-reviewed [52,115-118] and 8 gray literature [100,103,106,109,112-114,119] studies. The outcomes were evaluated through various methods, including RCTs (n=2), retrospective observational studies (n=2), qualitative studies (n=1), mixed studies (n=6), and cross-sectional surveys (n=2). One study was from Australia, 5 were from the United States, and 7 were from Canada. In addition to EHR and portal data, surveys, interviews, and focus groups were the most common sources of data. The studies analyzed the capacity of patient portals to increase vaccination rates, equity in access to timely care, and population empowerment.

##### Increase in Population Outreach

The RCTs examined the effectiveness of patient portals in improving influenza vaccination rates [115,117]. Although influenza infections have the potential to lead to serious health issues and increased access to health care services, vaccination rates continue to be low, necessitating the need for innovative outreach interventions to remind and encourage citizens to get the shot. As EHRs deliver real-time data identification, tethered patient portals were seen to have the potential to identify unvaccinated populations and enable implementation of portal-based cost-effective interventions. Cutrona et al [115] found a small but statistically significant improvement in the completion of influenza vaccination among portal users, especially by patients who opened reminder messages sent through the portal. Although a very small proportion (0.3%) of patients accessed the various influenza educational materials, Szilagyi et al [117] established a correlation between the higher numbers of reminders that led to higher vaccination rates by portal users. The portal reminders had a small, statistically significant effect on increasing rates among adults aged from 18 to 64 years, male patients, non-Hispanic patients, and those not vaccinated in the previous 2 years.

##### Decrease in Disparities Related to Access of Care Services

Foster et al [118] found that there were existing disparities between patient groups related to health care information access in emergency departments. African Americans and Hispanics had the lowest portal use rates, which led to disparities in medical information access. In their retrospective study, Lyles et al [116] reported a significant improvement in statin adherence regardless of race and ethnicity once patients increased portal use. The authors concluded that portal use could improve various health behaviors. Similarly, another study found that because of the ability to request and receive prescription renewals through a portal, patients did not need to travel, arrange care, or take time off work, which increased medication adherence and decreased wait time (74%) [112]. Another study concluded that *if only affluent, well-educated patients can access portals and understand them, then these technologies could potentially worsen health disparities* as one of the factors contributing to disparities in access were the decisions by providers to selectively offer access [100].

##### Improvement in Quality of Care

Two studies captured experiences of users who acknowledged that portals improved their quality of care and the ability to manage care because of information access [52,109]. However, because of low uptake by health care providers, they were uninformed about the portal. Two additional studies reported that Canadians felt more engaged and active, as the portal allowed them to have more informed discussions with their doctor [106,119]. Two evaluations found that access to health information contributed to easier access to services and acted as an *expansion of the standard 15 min consultation appointment* [113,114]. These studies demonstrated the readiness and willingness of patients to be more engaged in their health care. However, some apprehension was experienced, which could be elucidated by the *empowerment effect* related to web-based access to results and related information [103].

#### Health Care Workforce

In total, 18 studies [40-42,50,51,54,55,57,64,66,82,87,120-125] and 3 reports [114,126,127] addressed the health care workforce perspective through various methods (mixed methods, n=7; prospective observational, n=2; qualitative, n=6; and surveys, n=6), with a focus on conducting formative and process evaluations. A total of 11 studies were conducted in the United States, 5 in Sweden, 4 in Canada, and 1 in China. They explored health care provider experience with patient access to medical records, laboratory and diagnostic results, secure messaging, and uploading of images and symptoms. To gather data, focus groups, semistructured interviews, and surveys were used. Many of the studies used a combination of different data collection methods.

##### Impact of Patients Accessing Medical Records

Although health care providers generally agreed with the idea of patients having access to their information, they expressed concerns around patients’ understanding of the information contained in the medical record, especially access to laboratory or diagnostic test results [50,51,57,64,121,125]. One study reported an increase in the volume of inquiries and appointments due to patients not understanding the information. Another study found that providers had to spend more time reassuring patients after they read their records and medical notes [54]. A different study found that the majority of both physicians and nurses believed medical notes were confusing for patients, which had led to worry and increased contact between providers and patients [57]. With the intent to decrease patient confusion, few studies reflected on the aspect that portals had shifted how charting was done within the settings that offered access [42,54,66].

Many studies concluded that the health care workforce had a direct impact on portal adoption and utilization by patients [55,66,82,126]. Although paper access to medical records had always been obtainable and not seen as contentious, real-time access had been associated with concerns about privacy and security and led to discontent and low uptake by providers [120,123,124].

Only one study documented increased levels of threats and violence reported by staff from patients with access to their health records [40]. Staff reported that this was due to disagreement with the information in the medical record; however, the authors did not find increased incidents [40].

##### Impact on Workflow

Another concern was the workflow impact due to portal implementation. In one study, participating providers indicated that the portal implementation did not have a negative impact on their salary; however, 43% of the same participants believed that the portal increased their workload [41]. Similar findings were presented by Cajander et al [42], as the nurses in the study described how patients called and sent messages for the same question, which led to increased workload because of duplication of services. Furthermore, patients came prepared with more informed questions which led to *more in-depth discussions* that took additional unplanned appointment time [42].

Another study captured the experiences of providers who described cases in which patients contacted them for abnormalities that were clinically insignificant, thereby increasing the workload [125]. Vydra et al [66] compared provider reported time dedicated to portal-related duties with the administrative data captured by the system, as providers in their study reported spending an average of 12.5 hours per week logged into the portal; however, institutional records indicated an average of 8.2 hours per week.

##### Impact of Bidirectional Secure Messaging

Several studies captured the apprehensions of providers due to secure messaging [122,124]. In these studies, health care providers articulated the lack of clarity around appropriate ways to communicate via a portal as patients had expectations to receive immediate responses to their inquires [124]. Lieu et al [122] reported provider anxiety because of the lack of volume restrictions on electronic messages and their coping strategies to timing their responses to patient messages. Another study found that messaging increased work outside normal work hours [114].

A Canadian study found that providers considered to be early adopters indicated that secure messaging improved communication and interactions between themselves and their patients [114].

##### Virtual Care

One study reported that patient portals were effective for postoperative care, as patients uploaded images instead of scheduling face-to-face visits [87]. In this study, surgeons reported that web-based and clinic visits were equally effective for 68% (34/50) of patients.

#### Health System Perspective (Reduced Per Capita Cost of Health Care)

The health system perspective was explored by 15 peer-reviewed [45,48,49,59,65,69-71,128-134] and 3 gray literature [106,109,135] studies, which focused on the impact of patient portals on the potential for reducing costs. The studies varied in data collection approaches (prospective observational, n=4; retrospective observational, n=9; survey methods, n=2; and mixed methods, n=3), and the evaluated portal components (portal in general, n=11; viewing laboratory results, n=1; OpenNotes, n=1; secure messaging, n=4; appointments, n=2; and care plan, n=1). A total of 14 studies were from the United States, and there was 1 from Spain, Finland, and Canada. In all studies, EHR administrative data were used to compare pre- and postintervention inputs and outputs. In addition to the EHR, portal administrative data, workbench, interviews, charts, and tools that measured patient activation, quality of life, self-efficacy, and experience were applied. The studies explored whether implementation and subsequent adoption of a patient portal reduced costs in the evaluated areas. The included studies also explored whether patient portal use led to a decrease in 30-day all-cause readmission rates, clinical services utilization, no-show appointments, and the number of traditional encounters (office visits and telephone calls).

##### Decrease in No-Show Appointments

Studies evaluating the possible impact of patient portals on no-show appointments compared the pre-post no-show appointment rates and established that portal users had a lower rate of no-show appointments compared with nonusers; however, there was no difference in appointment cancellation rates [48,69,70,129,130]. Mendel et al [130] found portal enrollment increased once the clinic staff promoted the portal as a tool for appointment reminders, which was also associated with increased patient satisfaction [130]. However, once the portal started experiencing technical glitches, the decrease was maintained for only a short period of time. Zhong et al [69] found that no-show rates for portal users were 30% less than for nonusers and that frequent users of secure messaging and viewing laboratory test functions had the largest reduction in no-show rates. Similar findings were captured by Graham et al [48], who found a 53% relative reduction in no-show appointments in the clinics that piloted a patient portal, and the Ontario Shores Centre for Mental Health Sciences showed that portal users missed 18% of total appointments compared with nonportal users, who missed 20% of total appointments [135].

##### Impact on Office Visits and Telephone Calls

Several studies [128,132-134] evaluated the impact of secure messaging on office visits, telephone rates, and hospital readmissions. These studies had similar findings regardless of the applied methodology. Dexter et al [132] hypothesized that an increase in secure messaging use would decrease telephone call rates; however, the authors found that as messages increased, so did the number of telephone calls to the clinics. Similarly, Bryan et al [133] found that patients who sent more messages had higher clinical and phone encounters than those who did not send any. This impacted the workflow and the ability of care settings to handle the influx of visits and calls. Plate et al [128] demonstrated that if patients sent >2 messages and the clinic response rate was <75%, this significantly increased 90-day readmissions and 90-day emergency department visits.

##### Impact on Admission and Readmission Rates and Emergency Department Visits

Four studies [45,49,59,65] evaluated whether patient portals had the capacity to decrease readmission rates, emergency department visits, and hospital admissions. Nicolas et al [59] found a decrease in the rate of hospital admissions (5.28/10,000 per year) and 30-day all-cause readmissions (5.20/10,000 per year), whereas no increase in outpatient visit rates in the postintervention period of the portal implementation [59]. Similarly, Sorondo et al [65] concluded that there was a decrease in emergency department visits by at least 21% per 1000 and hospital admission rates by at least 38% per 1000. Although Nicolas et al [59] and Sorondo et al [65] found a decrease, Dumitrascu et al [45] and Griffin et al [49] concluded that portals users were associated with higher use of medical services and higher hospital readmissions.

##### Impact on Health Care Utilization

Four studies examined whether increased portal use decreased care utilization. Leveille et al [131] could not find any correlation between viewing medical notes and the number of appointments, whereas Zhou et al [71] concluded that viewing laboratory test results led to increased clinic visits and telephone calls. In their study, eHealth Saskatchewan reported that the number of primary care visits decreased because of patients using the portal [109]. This was also found in patient self-reported surveys. In their report, Canada Health Infoway reported an increase in e-visits because of patients having access to technologies providing e-service opportunities [106].

### Mapping According to the BE Framework

Altogether, 77 peer-reviewed studies were mapped on the expanded version of the BE framework. The mapping was done by subdimension to create a more precise representation about the areas that are currently explored when studying patient portals. Most of the studies evaluated more than one subdimension. An overview of the number of studies per subdimension is shown in Multimedia Appendix 7. The numbers for each subdimension represent the number of studies that addressed a particular component.

The authors of 48 out of the 77 studies explored the ability of patient and providers to access services, followed by use behavior/pattern (n=48), user satisfaction (n=34), self-reported use (n=31), patient/caregiver participation (n=30), knowledge, attitude, perception, decision confidence, compliance (n=29), and ease of use (n=26).

The least measured changes related to security (n=1); data quality improvement, reduced loss/paper, and transcription errors (n=2); responsiveness (n=3), barriers, training, organizational support, time-to-evaluation, lessons, success factors, project management, leadership, and costs (n=4); functionality (n=5); and performance (n=6).

Detailed mapping of each study per BE framework subdimension is presented in Multimedia Appendix 7 [27,40-99,115-118,120-125,128-134].

## Discussion

### Principal Findings

The implementation of technologies such as patient portals is a convoluted process with ambiguous returns on investment. Frequently, implementation planning is intertwined with optimism related to *if we offer it, they will come*. However, the current realities show that the planning and implementation stages are based on very little preparation related to visualizing the identified need for the technology and the issues that it is trying to amend. On the basis of the results from this scoping review, several gaps in the literature were identified. For each documented gap, summary recommendations are provided on how to improve the measuring impact of patient portals based on the QA and BE frameworks.

#### Lack of Studies With Multidimensional Impact Evaluation Strategies

Although this study was based on comprehensive inclusion criteria, there were no peer-reviewed or non–peer-reviewed studies that measured impact based on all dimensions from the QA or BE frameworks. Although several reports describing the use of the BE framework were included, indicators and outcomes measured still focused on certain dimensions of the frameworks rather than most or all dimensions. From the 96 included studies, the most evaluated number of dimensions was 2. In the gray literature, however, studies often evaluated 3 of the 4 dimensions. When applying the same mapping process to included studies through the extended version of the BE framework [35], the greatest number of evaluated dimensions was 9 [123,125].

Multidimensional evaluation strategies require that research studies capture the patient experience (ie, patient satisfaction, patient engagement, convenience of care, care plan compliance, patient to care team ratio, and access to care), which is related to the population experience (ie, improved health outcomes, compliance with standards of care, insight about population health, and quality of life while reducing complications, mortality rate, hospital admissions, and emergency department visits). As patient and population experiences are interconnected with the health care provider experience (ie, reduced redundant tasks, burnout, and turnover rate while improving resource utilization, satisfaction, and provider-patient relationship), they need to be measured along with the health system experience (ie, reduced cancellations, staff costs, cost per patient, costs because of readmission and emergency department visits, length of hospital stay while developing improved opportunities for reimbursements). These outcomes also fit within the BE framework; however, measures of the impact in terms of system quality (ie, functionality, performance, and security), information quality (ie, content and availability), and service quality (ie, responsiveness) need to be developed and added.

#### Lack of Studies Based on Suitable Methodology and Sample Size to Evaluate Patient Portal Technology

A substantial gap in the literature was the lack of prospective longitudinal studies with large samples. There were a few prospective studies [55,65,85] that followed patients between 7 and 12 months; however, the sample sizes were small (between 20 and 94 participants). Nearly all studies that evaluated the patient perspective acknowledged that the study samples were made up of early adopters and individuals from the higher socioeconomic spectrum. Study limitations were the low study response and participation rates, the lack of ethically and racially diverse participants, and the lack of nonusers (patients and providers) perspectives.

Surveys used to measure satisfaction were *newly developed* or *developed based on previous studies*, and thus, there was little evidence of reliability and validity. The Canada Health Infoway System and Use Survey [136] was used by all BE studies; however, their reliability and validity were unclear.

In addition, during the review of the included studies, there were inconsistencies between terms such as *indicators*, *outcomes*, *measures*, *tools*, and *net benefits* as they relate to measuring patient portal impact. Although many studies used BE framework terminology, the concepts were applied in different ways.

Longitudinal studies of the impact of patient portals on patients would provide more real-world data about how users of portals interact and what potentially meaningful changes are needed. These types of studies could provide evidence about cause-and-effect relationships, which remain minimally explored from the standpoint of portal use and quality of care, satisfaction, communication, and health outcomes. Size and diversity in the patient and provider samples are key to envisaging solutions that would lead to use and eventually improve value-based care. In addition, using validated surveys would ensure that the right things are measured correctly. Measuring satisfaction with technology needs to encompass elements such as preference, proficiency, and performance.

#### Lack of Recognition and Evidence Utilization From Organizational and Health System–Level Internal Impact Evaluations of Patient Portals

During the gray literature search, many organizational and health system patient portal evaluation reports were identified. However, when efforts were made to find the corresponding peer-reviewed publications, none were found. As these BE reports (all from Canadian jurisdictions) measured the impact in several BE framework dimensions, it would be helpful for these studies and their findings to be acquired through peer-reviewed journals. Such publications would provide evidence on how to evaluate patient portal impact and would be shared more extensively. Furthermore, real-world impact evaluations would guide investigators in directing research that is deemed important by organizations and systems that implement health information technologies.

#### Lack of Operative Recommendations Based on Study Findings

Frequently, findings were not followed up by concrete recommendations as to what was needed to rectify the documented obstacles. For example, patient and provider satisfaction were considered important outcomes and measured through interviews or surveys; however, by stating that patients reported high satisfaction or providers reported low satisfaction, the studies did not elaborate on what the satisfaction levels meant in terms of changes or modifications. Are measures such as medium-to-high or high satisfaction enough to conclude that the evaluated patient portal was effective and should be maintained? Through this scoping review, the evidence showed that suggestions for change were based on technical or user change (ie, accessibility or increased provider use). The current evidence warrants recommendations for changes that can be effectively implemented and evaluated but require system change.

#### Lack of Use of Patient Self-Reported Health Outcomes

Only one study [65] reported using a patient self-reported health outcome tool (EuroQol-visual analogue scales [EQ-VAS]). Despite studies [43,44,46,53,96] showing correlations between chronic conditions, medication adherence, and use of patient portals, there is a lack of application and use of patient self-reported health outcome tools in patient portal studies that measure impact [137]. Patient self-reported outcomes have the capacity to improve the quality of patient care; however, they are very difficult to measure or capture through interviews. These types of outcome tools are cost-effective and can easily be integrated within the patient’s portal structure. As patient portals are implemented in diverse settings, the use of these types of tools would provide the ability to determine the unique threshold levels and plan for patient portal intervention strategies that would be more effective and appropriate for each setting. Measuring patient portal impact by incorporating patient self-reported health outcome tools would allow for condition-specific portal enhancements with the possibility of increased adoption and use.

### Limitations

This scoping review included some literature that was not peer reviewed, and the strength of the evidence in these studies was not evaluated. First, the authors did not contact any research experts to help identify other gray literature. Second, only English language articles were included, which limited the databases and search terms used. For this reason, although a large number of citations were included, some relevant articles may have been missed. Third, as customary, scoping reviews do not quantitatively synthesize the findings; therefore, statistical conclusions may be drawn from the results regarding effects, statistical significance, or bias evaluation. Finally, study screening and selection are subjective processes. Although a high level of agreement was achieved, there was a reliance on judgment to include and exclude studies.

### Conclusions

Despite extensive and existing research in the area of patient portals, the evidence from this scoping review suggests that impact research is available; however, it lacks multidimensionality. The QA and BE frameworks provided guidance in identifying the gaps in the current literature by providing a way to show how impact was assessed. This study highlights the need to appropriately plan how impact will be assessed and how the findings will be translated into effective adaptations. If the *how* and *what* are not properly planned, the generalizability of patient portal studies will continue to elude researchers and implementation teams.

## Appendix

Multimedia Appendix 1 Scoping review search strategy.

Multimedia Appendix 2 Synthesis of included studies.

Multimedia Appendix 3 Quadruple Aim patient perspective table.

Multimedia Appendix 4 Quadruple Aim population perspective table.

Multimedia Appendix 5 Quadruple Aim healthcare provider perspective table.

Multimedia Appendix 6 Quadruple Aim Health system perspective table.

Multimedia Appendix 7 Mapping the peer-reviewed studies according to the Benefits Evaluation Framework.

## Abbreviations

BE: Benefits Evaluation
CINAHL: Cumulative Index of Nursing and Allied Health Literature
EHR: electronic health record
HbA_1c_: glycated hemoglobin
PRESS: peer review of electronic search strategies
PRISMA: Preferred Reporting Items for Systematic reviews and Meta-Analyses
QA: Quadruple Aim
RCT: randomized controlled trial
