# Supplementary material for: Capturing the Impact of Patient Portals Based on the Quadruple Aim and Benefits Evaluation Frameworks: Scoping Review
Source: J Med Internet Res. 2020 Dec 8;22(12):e24568. doi: 10.2196/24568 (PMC7755541; doi:10.2196/24568)
Supplement: Multimedia Appendix 1 [file jmir_v22i12e24568_app1.docx]

# Scoping review search strategy

Patient Portals

Final Strategy

2020 Jun 8

## Ovid Multifile

Database: Ovid MEDLINE(R) ALL <1946 to June 05, 2020>, Embase <1974 to 2020 June 05>, APA PsycInfo <1806 to June Week 1 2020>

Search Strategy:

--------------------------------------------------------------------------------

1 Patient Portals/ (175043)

2 (patient? adj2 (portal or portals)).tw,kf. (11738)

3 Electronic Health Records/ (35402)

4 limit 3 to yr="2010-2016" (15491)

5 4 and (portal or portals).tw,kf. (349)

6 4 and (patient* adj2 access*).tw,kf. (305)

7 4 and (personal* adj2 access*).tw,kf. (40)

8 ((web or web-based or web-site or website or internet or online or www or cyber*) adj3 (portal or portals)).tw,kf. (5756)

9 ((health record? or EHR or EHRs or PHR or PHRs) adj3 (portal or portals)).tw,kf. (417)

10 ((health record? or EHR or EHRs or PHR or PHRs) adj3 (patient* adj2 access*)).tw,kf. (328)

11 ((health record? or EHR or EHRs or PHR or PHRs) adj3 (person* adj2 access*)).tw,kf. (80)

12 ((medical record? or EMR or EMRs) adj3 (portal or portals)).tw,kf. (126)

13 ((medical record? or EMR or EMRs) adj3 (patient* adj2 access*)).tw,kf. (522)

14 ((medical record? or EMR or EMRs) adj3 (person* adj2 access*)).tw,kf. (36)

15 (clinical record? adj3 (portal or portals)).tw,kf. (2)

16 (clinical record? adj3 (patient* adj2 access*)).tw,kf. (28)

17 (clinical record? adj3 (person* adj2 access*)).tw,kf. (0)

18 ((health information or medical information or clinical information) adj3 (portal or portals)).tw,kf. (175)

19 ((health information or medical information or clinical information) adj3 (patient* adj2 access*)).tw,kf. (359)

20 ((health information or medical information or clinical information) adj3 (person* adj2 access*)).tw,kf. (177)

21 ((health data or medical data or clinical data) adj3 (portal or portals)).tw,kf. (66)

22 ((health data or medical data or clinical data) adj3 (patient* adj2 access*)).tw,kf. (86)

23 ((health data or medical data or clinical data) adj3 (person* adj2 access*)).tw,kf. (43)

24 health portal?.tw,kf. (341)

25 ((ehealth or e-health or mhealth or m-health or mobile health) adj3 (portal or portals)).tw,kf. (105)

26 ((ehealth or e-health or mhealth or m-health or mobile health) adj3 (patient* adj2 access*)).tw,kf. (17)

27 ((ehealth or e-health or mhealth or m-health or mobile health) adj3 (person* adj2 access*)).tw,kf. (6)

28 (resource? adj3 (portal or portals)).tw,kf. (221)

29 Electronic Health Records/ (35402)

30 Health Records, Personal/ (167153)

31 Internet/ (209452)

32 Health Services Accessibility/ (121030)

33 Information Seeking Behavior/ (5671)

34 Patient Access to Records/ (14759)

35 Patient-Centered Care/ (197819)

36 Patient Participation/ (54970)

37 Physician-Patient Relations/ (75194)

38 Self Care/ (95439)

39 Self-Management/ (55738)

40 (29 or 30) and (31 or 32 or 33 or 34 or 35 or 36 or 37 or 38 or 39) (12743)

41 or/1-2,5-28,40 [PATIENT PORTALS] (196090)

42 exp Animals/ not Humans/ (16838428)

43 41 not 42 [ANIMAL-ONLY REMOVED] (135426)

44 43 use medall [MEDLINE RECORDS] (9169)

45 (patient? adj2 (portal or portals)).tw,kw. (11739)

46 ((web or web-based or web-site or website or internet or online or www or cyber*) adj3 (portal or portals)).tw,kw. (5763)

47 ((health record? or EHR or EHRs or PHR or PHRs) adj3 (portal or portals)).tw,kw. (417)

48 ((health record? or EHR or EHRs or PHR or PHRs) adj3 (patient* adj2 access*)).tw,kw. (330)

49 ((health record? or EHR or EHRs or PHR or PHRs) adj3 (person* adj2 access*)).tw,kw. (81)

50 ((medical record? or EMR or EMRs) adj3 (portal or portals)).tw,kw. (126)

51 ((medical record? or EMR or EMRs) adj3 (patient* adj2 access*)).tw,kw. (526)

52 ((medical record? or EMR or EMRs) adj3 (person* adj2 access*)).tw,kw. (37)

53 (clinical record? adj3 (portal or portals)).tw,kw. (2)

54 (clinical record? adj3 (patient* adj2 access*)).tw,kw. (28)

55 (clinical record? adj3 (person* adj2 access*)).tw,kw. (0)

56 ((health information or medical information or clinical information) adj3 (portal or portals)).tw,kw. (176)

57 ((health information or medical information or clinical information) adj3 (patient* adj2 access*)).tw,kw. (359)

58 ((health information or medical information or clinical information) adj3 (person* adj2 access*)).tw,kw. (177)

59 ((health data or medical data or clinical data) adj3 (portal or portals)).tw,kw. (66)

60 ((health data or medical data or clinical data) adj3 (patient* adj2 access*)).tw,kw. (87)

61 ((health data or medical data or clinical data) adj3 (person* adj2 access*)).tw,kw. (44)

62 health portal?.tw,kw. (349)

63 ((ehealth or e-health or mhealth or m-health or mobile health) adj3 (portal or portals)).tw,kw. (106)

64 ((ehealth or e-health or mhealth or m-health or mobile health) adj3 (patient* adj2 access*)).tw,kw. (19)

65 ((ehealth or e-health or mhealth or m-health or mobile health) adj3 (person* adj2 access*)).tw,kw. (7)

66 (resource? adj3 (portal or portals)).tw,kw. (221)

67 electronic health record/ (36611)

68 electronic medical record/ (74298)

69 electronic patient record/ (2299)

70 Internet/ (209452)

71 information seeking/ (7117)

72 patient right/ (22384)

73 exp health care access/ (66868)

74 patient participation/ (54970)

75 doctor patient relationship/ (2962)

76 self care/ (95439)

77 self help/ (17607)

78 (67 or 68 or 69) and (70 or 71 or 72 or 73 or 74 or 75 or 76 or 77) (5193)

79 or/45-66,78 [PATIENT PORTALS] (23281)

80 exp animal/ or exp animal experimentation/ or exp animal model/ or exp animal experiment/ or nonhuman/ or exp vertebrate/ (50995912)

81 exp human/ or exp human experimentation/ or exp human experiment/ (39478597)

82 80 not 81 (11518911)

83 79 not 82 [ANIMAL-ONLY REMOVED] (22937)

84 83 use oemezd [EMBASE RECORDS] (14113)

85 (patient? adj2 (portal or portals)).tw,id. (11648)

86 ((web or web-based or web-site or website or internet or online or www or cyber*) adj3 (portal or portals)).tw,id. (5726)

87 ((health record? or EHR or EHRsor PHR or PHRs) adj3 (portal or portals)).tw,id. (368)

88 ((health record? or EHR or EHRs or PHR or PHRs) adj3 (patient* adj2 access*)).tw,id. (325)

89 ((health record? or EHR or EHRs or PHR or PHRs) adj3 (person* adj2 access*)).tw,id. (80)

90 ((medical record? or EMR or EMRs) adj3 (portal or portals)).tw,id. (126)

91 ((medical record? or EMR or EMRs) adj3 (patient* adj2 access*)).tw,id. (521)

92 ((medical record? or EMR or EMRs) adj3 (person* adj2 access*)).tw,id. (36)

93 (clinical record? adj3 (portal or portals)).tw,id. (2)

94 (clinical record? adj3 (patient* adj2 access*)).tw,id. (28)

95 (clinical record? adj3 (person* adj2 access*)).tw,id. (0)

96 ((health information or medical information or clinical information) adj3 (portal or portals)).tw,id. (175)

97 ((health information or medical information or clinical information) adj3 (patient* adj2 access*)).tw,id. (358)

98 ((health information or medical information or clinical information) adj3 (person* adj2 access*)).tw,id. (177)

99 ((health data or medical data or clinical data) adj3 (portal or portals)).tw,id. (66)

100 ((health data or medical data or clinical data) adj3 (patient* adj2 access*)).tw,id. (86)

101 ((health data or medical data or clinical data) adj3 (person* adj2 access*)).tw. (43)

102 health portal?.tw,id. (340)

103 ((ehealth or e-health or mhealth or m-health or mobile health) adj3 (portal or portals)).tw,id. (105)

104 ((ehealth or e-health or mhealth or m-health or mobile health) adj3 (patient* adj2 access*)).tw,id. (17)

105 ((ehealth or e-health or mhealth or m-health or mobile health) adj3 (person* adj2 access*)).tw,id. (6)

106 (resource? adj3 (portal or portals)).tw,id. (221)

107 Electronic Health Records/ (35402)

108 Internet/ (209452)

109 exp Information Seeking/ (10059)

110 Client Participation/ (2229)

111 Self-Management/ (55738)

112 107 and (108 or 109 or 110 or 111) (1178)

113 or/85-106,112 [PATIENT PORTALS] (19482)

114 113 use medall,oemezd (18564)

115 113 not 114 [PSYCINFO RECORDS] (918)

116 44 or 84 or 115 [ALL DATABASES] (24200)

117 limit 116 to yr="2018-current" (5041)

118 remove duplicates from 117 (3571)

119 limit 116 to yr="2015-2017" (5256)

120 remove duplicates from 119 (3868)

121 limit 116 to yr="2011-2014" (5684)

122 remove duplicates from 121 (4155)

123 limit 116 to yr="2000-2010" (4956)

124 remove duplicates from 123 (3371)

125 116 not (117 or 119 or 121 or 121 or 123) (3263)

126 remove duplicates from 125 (1942)

127 118 or 120 or 122 or 124 or 126 [TOTAL UNIQUE RECORDS] (16907)

128 127 use medall [MEDLINE UNIQUE RECORDS] (9071)

129 127 use oemezd [EMBASE RECORDS] (7302)

130 127 not (128 or 129) [PSYCINFO RECORDS] (534)

***************************

## CINAHL

| # | Query | Limiters/Expanders | Results |
| --- | --- | --- | --- |
| S40 | S37 OR S38 | Limiters - Exclude MEDLINE records  Expanders - Apply equivalent subjects  Search modes - Find all my search terms | 3,494 |
| S39 | S37 OR S38 | Expanders - Apply equivalent subjects  Search modes - Find all my search terms | 6,292 |
| S38 | S1 OR S2 OR S3 OR S4 OR S5 OR S6 OR S7 OR S8 OR S9 OR S10 OR S11 OR S12 OR S13 OR S14 OR S15 OR S16 OR S17 OR S18 OR S19 OR S20 OR S21 OR S22 OR S23 | Expanders - Apply equivalent subjects  Search modes - Find all my search terms | 3,526 |
| S37 | S35 AND S36 | Expanders - Apply equivalent subjects  Search modes - Find all my search terms | 3,045 |
| S36 | S26 OR S27 OR S28 OR S29 OR S30 OR S31 OR S32 OR S33 OR S34 | Expanders - Apply equivalent subjects  Search modes - Find all my search terms | 264,761 |
| S35 | S24 OR S25 | Expanders - Apply equivalent subjects  Search modes - Find all my search terms | 27,293 |
| S34 | (MH "Self-Management") | Expanders - Apply equivalent subjects  Search modes - Find all my search terms | 744 |
| S33 | (MH "Self Care") | Expanders - Apply equivalent subjects  Search modes - Find all my search terms | 42,813 |
| S32 | (MH "Physician-Patient Relations") | Expanders - Apply equivalent subjects  Search modes - Find all my search terms | 34,259 |
| S31 | (MH "Consumer Participation") | Expanders - Apply equivalent subjects  Search modes - Find all my search terms | 20,894 |
| S30 | (MH "Patient Centered Care") | Expanders - Apply equivalent subjects  Search modes - Find all my search terms | 32,860 |
| S29 | (MH "Patient Access to Records") | Expanders - Apply equivalent subjects  Search modes - Find all my search terms | 1,007 |
| S28 | (MH "Information Seeking Behavior") | Expanders - Apply equivalent subjects  Search modes - Find all my search terms | 4,684 |
| S27 | (MH "Health Services Accessibility+") | Expanders - Apply equivalent subjects  Search modes - Find all my search terms | 92,116 |
| S26 | (MH "Internet") | Expanders - Apply equivalent subjects  Search modes - Find all my search terms | 52,241 |
| S25 | (MH "Medical Records, Personal") | Expanders - Apply equivalent subjects  Search modes - Find all my search terms | 1,267 |
| S24 | (MH "Electronic Health Records") | Expanders - Apply equivalent subjects  Search modes - Find all my search terms | 26,364 |
| S23 | TI ( resource# N3 (portal or portals) ) OR AB ( resource# N3 (portal or portals) ) | Expanders - Apply equivalent subjects  Search modes - Find all my search terms | 63 |
| S22 | TI ( (ehealth or "e-health" or mhealth or "m-health" or "mobile health") N3 (personal* N2 access*) ) OR AB ( (ehealth or "e-health" or mhealth or "m-health" or "mobile health") N3 (personal* N2 access*) ) | Expanders - Apply equivalent subjects  Search modes - Find all my search terms | 5 |
| S21 | TI ( (ehealth or "e-health" or mhealth or "m-health" or "mobile health") N3 (patient* N2 access*) ) OR AB ( (ehealth or "e-health" or mhealth or "m-health" or "mobile health") N3 (patient* N2 access*) ) | Expanders - Apply equivalent subjects  Search modes - Find all my search terms | 11 |
| S20 | TI ( (ehealth or "e-health" or mhealth or "m-health" or "mobile health") N3 (portal or portals) ) OR AB ( (ehealth or "e-health" or mhealth or "m-health" or "mobile health") N3 (portal or portals) ) | Expanders - Apply equivalent subjects  Search modes - Find all my search terms | 21 |
| S19 | TI ( "health portal" or "health portals ) OR AB ( "health portal" or "health portals ) | Expanders - Apply equivalent subjects  Search modes - Find all my search terms | 1,560 |
| S18 | TI ( ("health data" or "medical data" or "clinical data") N3 (personal* N2 access*) ) OR AB ( ("health data" or "medical data" or "clinical data") N3 (personal* N2 access*) ) | Expanders - Apply equivalent subjects  Search modes - Find all my search terms | 12 |
| S17 | TI ( ("health data" or "medical data" or "clinical data") N3 (patient* N2 access*) ) OR AB ( ("health data" or "medical data" or "clinical data") N3 (patient* N2 access*) ) | Expanders - Apply equivalent subjects  Search modes - Find all my search terms | 38 |
| S16 | TI ( ("health data" or "medical data" or "clinical data") N3 (portal or portals) ) OR AB ( ("health data" or "medical data" or "clinical data") N3 (portal or portals) ) | Expanders - Apply equivalent subjects  Search modes - Find all my search terms | 14 |
| S15 | TI ( ("health information" or "medical information" or "clinical information") N3 (personal* N2 access*) ) OR AB ( ("health information" or "medical information" or "clinical information") N3 (personal* N2 access*) ) | Expanders - Apply equivalent subjects  Search modes - Find all my search terms | 70 |
| S14 | TI ( ("health information" or "medical information" or "clinical information") N3 (patient* N2 access*) ) OR AB ( ("health information" or "medical information" or "clinical information") N3 (patient* N2 access*) ) | Expanders - Apply equivalent subjects  Search modes - Find all my search terms | 154 |
| S13 | TI ( ("health information" or "medical information" or "clinical information") N3 (portal or portals) ) OR AB ( ("health information" or "medical information" or "clinical information") N3 (portal or portals) ) | Expanders - Apply equivalent subjects  Search modes - Find all my search terms | 67 |
| S12 | TI ( ("clinical record" or "clinical records") N3 (personal* N2 access*) ) OR AB ( ("clinical record" or "clinical records") N3 (personal* N2 access*) ) | Expanders - Apply equivalent subjects  Search modes - Find all my search terms | 0 |
| S11 | TI ( ("clinical record" or "clinical records") N3 (patient* N2 access*) ) OR AB ( ("clinical record" or "clinical records") N3 (patient* N2 access*) ) | Expanders - Apply equivalent subjects  Search modes - Find all my search terms | 6 |
| S10 | TI ( ("clinical record" or "clinical records") N3 (portal or portals) ) OR AB ( ("clinical record" or "clinical records") N3 (portal or portals) ) | Expanders - Apply equivalent subjects  Search modes - Find all my search terms | 1 |
| S9 | TI ( ("medical record" or "medical records" or EMR or EMRs) N3 (personal* N2 access*) ) OR AB ( ("medical record" or "medical records" or EMR or EMRs) N3 (personal* N2 access*) ) | Expanders - Apply equivalent subjects  Search modes - Find all my search terms | 8 |
| S8 | TI ( ("medical record" or "medical records" or EMR or EMRs) N3 (patient* N2 access*) ) OR AB ( ("medical record" or "medical records" or EMR or EMRs) N3 (patient* N2 access*) ) | Expanders - Apply equivalent subjects  Search modes - Find all my search terms | 166 |
| S7 | TI ( ("medical record" or "medical records" or EMR or EMRs) N3 (portal or portals) ) OR AB ( ("medical record" or "medical records" or EMR or EMRs) N3 (portal or portals) ) | Expanders - Apply equivalent subjects  Search modes - Find all my search terms | 40 |
| S6 | TI ( ("health record" or "health records" or EHR or EHRs or PHR or PHRs) N3 (personal* N2 access*) ) OR AB ( ("health record" or "health records" or EHR or EHRs or PHR or PHRs) N3 (personal* N2 access*) ) | Expanders - Apply equivalent subjects  Search modes - Find all my search terms | 38 |
| S5 | TI ( ("health record" or "health records" or EHR or EHRs or PHR or PHRs) N3 (patient* N2 access*) ) OR AB ( ("health record" or "health records" or EHR or EHRs or PHR or PHRs) N3 (patient* N2 access*) ) | Expanders - Apply equivalent subjects  Search modes - Find all my search terms | 169 |
| S4 | TI ( ("health record" or "health records" or EHR or EHRs or PHR or PHRs) N3 (portal or portals) ) OR AB ( ("health record" or "health records" or EHR or EHRs or PHR or PHRs) N3 (portal or portals) ) | Expanders - Apply equivalent subjects  Search modes - Find all my search terms | 131 |
| S3 | TI ( (web or "web-based" or "web-site" or website or internet or online or www or cyber*) N3 (portal or portals) ) OR AB ( (web or "web-based" or "web-site" or website or internet or online or www or cyber*) N3 (portal or portals) ) | Expanders - Apply equivalent subjects  Search modes - Find all my search terms | 1,037 |
| S2 | TI ( patient# N2 (portal or portals) ) OR AB ( patient# N2 (portal or portals) ) | Expanders - Apply equivalent subjects  Search modes - Find all my search terms | 1,429 |
| S1 | (MH "Patient Portals") | Expanders - Apply equivalent subjects  Search modes - Find all my search terms | 88 |

## Web of Science

| # 6 | [21,018](http://apps.webofknowledge.com.login.ezproxy.library.ualberta.ca/summary.do?product=WOS&doc=1&qid=10&SID=7ArJU7LkLrGjlJpG55L&search_mode=CombineSearches&update_back2search_link_param=yes) | #5  OR  #4  OR  #3  OR  #2  OR  #1  Indexes=SCI-EXPANDED, SSCI, A&HCI, CPCI-S, CPCI-SSH, BKCI-S, BKCI-SSH, ESCI, CCR-EXPANDED, IC Timespan=All years |
| --- | --- | --- |
| # 5 | [1,530](http://apps.webofknowledge.com.login.ezproxy.library.ualberta.ca/summary.do?product=WOS&doc=1&qid=9&SID=7ArJU7LkLrGjlJpG55L&search_mode=GeneralSearch&update_back2search_link_param=yes) | **TOPIC:**  ((ehealth or "e-health" or mhealth or "m-health" or "mobile health")  NEAR/3  portal)  *OR*  **TOPIC:**  ((ehealth or "e-health" or mhealth or "m-health" or "mobile health")  NEAR/3  portals)  *OR*  **TOPIC:**  ((ehealth or "e-health" or mhealth or "m-health" or "mobile health")  NEAR/3  patient  access*)  *OR*  **TOPIC:**  ((ehealth or "e-health" or mhealth or "m-health" or "mobile health")  NEAR/3  personal  access*)  *OR*  **TOPIC:**  (resource* NEAR/3 (portal or portals) )  Indexes=SCI-EXPANDED, SSCI, A&HCI, CPCI-S, CPCI-SSH, BKCI-S, BKCI-SSH, ESCI, CCR-EXPANDED, IC Timespan=All years |
| # 4 | [2,163](http://apps.webofknowledge.com.login.ezproxy.library.ualberta.ca/summary.do?product=WOS&doc=1&qid=8&SID=7ArJU7LkLrGjlJpG55L&search_mode=GeneralSearch&update_back2search_link_param=yes) | **TOPIC:**  (("health data" or "medical data" or "clinical data")  NEAR/3  portal)  *OR*  **TOPIC:**  (("health data" or "medical data" or "clinical data")  NEAR/3  portals)  *OR*  **TOPIC:**  (("health data" or "medical data" or "clinical data")  NEAR/3  patient  access*)  *OR*  **TOPIC:**  (("health data" or "medical data" or "clinical data")  NEAR/3  personal  access*)  *OR*  **TOPIC:**  ("health portal" or "health portals")  Indexes=SCI-EXPANDED, SSCI, A&HCI, CPCI-S, CPCI-SSH, BKCI-S, BKCI-SSH, ESCI, CCR-EXPANDED, IC Timespan=All years |
| # 3 | [3,656](http://apps.webofknowledge.com.login.ezproxy.library.ualberta.ca/summary.do?product=WOS&doc=1&qid=4&SID=7ArJU7LkLrGjlJpG55L&search_mode=GeneralSearch&update_back2search_link_param=yes) | **TOPIC:**  (("clinical record" or "clinical records")  NEAR/3  portal)  *OR*  **TOPIC:**  (("clinical record" or "clinical records")  NEAR/3  portals)  *OR*  **TOPIC:**  (("clinical record" or "clinical records")  NEAR/3  patient  access*)  *OR*  **TOPIC:**  (("clinical record" or "clinical records")  NEAR/3  personal  access*)  *OR*  **TOPIC:**  (("health information" or "medical information" or "clinical information")  NEAR/3  portal)  *OR*  **TOPIC:**  (("health information" or "medical information" or "clinical information")  NEAR/3  portal)  *OR*  **TOPIC:**  (("health information" or "medical information" or "clinical information")  NEAR/3  patient  access*)  *OR*  **TOPIC:**  (("health information" or "medical information" or "clinical information")  NEAR/3  personal  access*)  Indexes=SCI-EXPANDED, SSCI, A&HCI, CPCI-S, CPCI-SSH, BKCI-S, BKCI-SSH, ESCI, CCR-EXPANDED, IC Timespan=All years |
| # 2 | [4,694](http://apps.webofknowledge.com.login.ezproxy.library.ualberta.ca/summary.do?product=WOS&doc=1&qid=7&SID=7ArJU7LkLrGjlJpG55L&search_mode=AdvancedSearch&update_back2search_link_param=yes) | TS=(("health records"  or  EHR  or  EHRs  or  PHR  or  PHRs)  NEAR/3  portal)  OR  TS=(("health records"  or  EHR  or  EHRs  or  PHR  or  PHRs)  NEAR/3  portals)  OR  TS=(("health records"  or  EHR  or  EHRs  or  PHR  or  PHRs)  NEAR/3  patient  access*)  OR  TS=(("health records"  or  EHR  or  EHRs  or  PHR  or  PHRs)  NEAR/3  personal  access*)  OR  TS=(("medical record"  or  "medical  records"  or  EMR  or  EMRs)  NEAR/3  portal)  OR  TS=(("medical record"  or  "medical  records"  or  EMR  or  EMRs)  NEAR/3  portals)  OR  TS=(("medical record"  or  "medical  records"  or  EMR  or  EMRs)  NEAR/3  patient  access*)  OR  TS=(("medical record"  or  "medical  records"  or  EMR  or  EMRs)  NEAR/3  personal  access*)  Indexes=SCI-EXPANDED, SSCI, A&HCI, CPCI-S, CPCI-SSH, BKCI-S, BKCI-SSH, ESCI, CCR-EXPANDED, IC Timespan=All years |
| # 1 | [12,642](http://apps.webofknowledge.com.login.ezproxy.library.ualberta.ca/summary.do?product=WOS&doc=1&qid=2&SID=7ArJU7LkLrGjlJpG55L&search_mode=GeneralSearch&update_back2search_link_param=yes) | **TOPIC:**  (patient* NEAR/2 (portal or portals) )  *OR*  **TOPIC:**  ((web or "web-based" or "web-site" or website or internet or online or www or cyber*)  NEAR/3  portal)  *OR*  **TOPIC:**  ((web or "web-based" or "web-site" or website or internet or online or www or cyber*)  NEAR/3  portals)  *OR*  **TOPIC:**  (("health record" or EHR or EHRs or PHR or PHRs)  NEAR/3  portal)  *OR*  **TOPIC:**  (("health record" or EHR or EHRs or PHR or PHRs)  NEAR/3  portals)  *OR*  **TOPIC:**  (("health record" or EHR or EHRs or PHR or PHRs)  NEAR/3  patient  access*)  *OR*  **TOPIC:**  (("health record" or EHR or EHRs or PHR or PHRs)  NEAR/3  personal  access*)  Indexes=SCI-EXPANDED, SSCI, A&HCI, CPCI-S, CPCI-SSH, BKCI-S, BKCI-SSH, ESCI, CCR-EXPANDED, IC Timespan=All years |
