# Supplementary material for: Capturing the Impact of Patient Portals Based on the Quadruple Aim and Benefits Evaluation Frameworks: Scoping Review
Source: J Med Internet Res. 2020 Dec 8;22(12):e24568. doi: 10.2196/24568 (PMC7755541; doi:10.2196/24568)
Supplement: Multimedia Appendix 2 [file jmir_v22i12e24568_app2.docx]

**Synthesis of included studies**

| **Author/s** | **Title** | **Publication Year** | **Country** | **Study design** | **Aim** |
| --- | --- | --- | --- | --- | --- |
| Agency for Healthcare Research and Quality | A National Web Conference on Effective Design and Use of Patient Portals and their Impact on Patient-Centered Care | 2017 | United States | Mixed method | To present evidence on the implementation of effective patient portals. |
| Akerstedt et al | On threats and violence for staff and patient accessible electronic health records | 2018 | Sweden | Survey method | To explore the possible of violence toward stuff due to patient access to their medical records. |
| Barrie Community Health Link | Project Review and Benefits Evaluation Final Report Consumer Health Solution for Patient and Provider Communication | 2016 | Canada | Mixed method | To understand the relationship with a chosen solution, the adoption of that solution, and the resulting impacts. |
| Brohman et al | Community Paramedicine Remote Patient Monitoring [CPRPM]: Benefits Evaluation & Lessons Learned | 2015 | Canada | Mixed method | To describe the findings from a home-based remote patient monitoring system that transmitted data about a patient’s health status from home to healthcare providers through the patient use of portal. |
| Bryan et al | Resource Utilization Among Portal Users Who Send Messages: A Retrospective Cohort Study | 2020 | United States | Retrospective method | To investigate the impact of secure messaging on office visits and telephone calls. |
| Bush et al | Physician Perception of the Role of the Patient Portal in Pediatric Health | 2017 | United States | Mixed method | To gain insights about physician perceptions regarding the importance of patient portals in the pediatric environment. |
| Cajander et al | Medical Records Online for Patients and Effects on the Work Environment of Nurses | 2018 | Sweden | Qualitative method | To gain insight in the perceptions of nurses about the impact of personal health records on their work. |
| Canada Health Infoway | Access to Digital Health Services 2019 Survey of Canadians Summary Report | 2019 | Canada | Survey method | To present the results of a survey about use and citizen interest in accessing their health information online and digitally enabled health services. |
| Canada Health Infoway | Annual Report 2018-2019 A new day in health care is coming. | 2019 | Canada | Survey method | To describe the activities undertaken by Canada Health Infoway. |
| Canada Health Infoway | Environmental Scan: Processes to enable adolescent access to personal health records | 2016 | Canada | Mixed method | To describe how adolescent access to digital health information is implemented. |
| Canada Health Infoway | Backgrounder - Positive Patient Experience Yields Health Care Benefits | 2017 | Canada | Survey method | To report the use and interest of citizens in accessing health information online. |
| Canada Health Infoway | Understanding the Current State of Patient Provided Digital Health Information [Know Me] | 2015 | Canada | Mixed method | To gain understanding about what information patients are sharing [or want to share] with their health care providers that would contribute to providers’ understanding of who they are as people, beyond who they are as patients. |
| Canada Health Infoway | Valuing Canadians’ secure access to their health information and digital health eservices | 2018 | Canada | Qualitative method | Synthesize outcomes generated by benefits evaluations conducted at multiple sites implementing PHRs and/or eServices in Canada, across different types of care settings. |
| Canada Health Infoway | Current and potential value of Canadians’ secure access to their health information and digital health eServices | 2017 | Canada | Survey method | To describe the findings from the 2017 patient and provider surveys undertaken by Canada Health Infoway. |
| Children's Hospital of Eastern Ontario | Epic EHR Program MyChart Consumer Health Solutions Benefits Evaluation Report [Pilot] | 2015 | Canada | Mixed method | To present the findings from the evaluation to capture and document implementation of a portal as part of an EHR system. |
| Chimowitz et al | Empowering Informal Caregivers with Health Information: OpenNotes as a Safety Strategy | 2018 | United States | Survey method | To investigate the impact of OpenNotes on caregivers. |
| Crotty et al | Prevalence and Risk Profile Of Unread Messages To Patients In A Patient Web Portal | 2015 | United States | Retrospective method | To determine the prevalence and risk of patients who do not read their secure messages. |
| Crouch et al | A pilot study to evaluate the magnitude of association of the use of electronic personal health records with patient activation and empowerment in HIV-infected veterans | 2015 | United States | Survey method | To investigate the patient characteristics, healthcare empowerment, patient activation, and satisfaction associated with access to patient portal in veterans living with HIV. |
| Cutrona et al | Improving Rates of Outpatient Influenza Vaccination Through EHR Portal Messages and Interactive Automated Calls: A Randomized Controlled Trial | 2018 | United States | Randomized Controlled trial | To examine the effectiveness of a patient portal in improving rates of influenza vaccination. |
| Dalal et al | Potential of an Electronic Health Record-Integrated Patient Portal for Improving Care Plan Concordance during Acute Care | 2019 | United States | Prospective method | To determine the levels of patient portal usage by oncology patents and their healthcare providers. |
| Denneson et al | Patients' Positive and Negative Responses to Reading Mental Health Clinical Notes Online | 2018 | United States | Survey method | To investigate the impact of OpenNotes among veterans receiving mental health care. |
| Devkota et al | Use of an Online Patient Portal and Glucose Control in Primary Care Patients with Diabetes | 2016 | United States | Retrospective method | To investigate the impact of patient portal use on the improvement of HbA1c in patients with type 2 diabetes in primary care settings. |
| Dexter et al | Patient-Provider Communication: Does Electronic Messaging Reduce Incoming Telephone Calls? | 2016 | United States | Retrospective method | To investigate the rate of secure messaging. |
| Dumitrascu et al | Patient portal use and hospital outcomes | 2018 | United States | Retrospective method | To examine the association between hospital outcomes of 30-day readmissions, inpatient |
| eHealth Saskatchewan | Citizen health information portal [CHIP]: benefits evaluation report | 2017 | Canada | Mixed method | To present the findings from the evaluation to capture and document implementation of a portal as part of an EHR system. |
| eHealth Saskatchewan | Defining empowerment and supporting engagement Saskatchewan patients and the eHealth Saskatchewan citizen health information portal [CHIP] | 2016 | Canada | Mixed method | To describe the findings from a qualitative exploration about the use and access of the Saskatchewan portal. |
| Esch et al | Engaging patients through OpenNotes: an evaluation using mixed methods | 2016 | United States | Mixed method | To gain insight in the experiences of patients’ viewing visit notes and the impact on the patient-provider relationship. |
| Federman et al | Patient and clinician perspectives on the outpatient after-visit summary: a qualitative study to inform improvements in visit summary design | 2017 | United States | Qualitative method | To investigate the impact of After Visit Summaries on patients. |
| Fiks et al | Parent-reported outcomes of a shared decision-making portal in asthma: a practice-based RCT | 2015 | United States | Randomized Controlled Trial | To explore the feasibility and impact of a patient portal on shared-decision making. |
| Fossa et al | OpenNotes and shared decision making: a growing practice in clinical transparency and how it can support patient-centered care | 2018 | United States | Survey method | To investigate the impact of OpenNotes on patients through a scoring system. |
| Foster et al | The Use of an Electronic Health Record Patient Portal to Access Diagnostic Test Results by Emergency Patients at an Academic Medical Center: Retrospective Study | 2019 | United States | Retrospective method | To determine the use of patient portals by patients in the emergency department. |
| Garry et al | Patient Experience With Notification of Radiology Results: A Comparison of Direct Communication and Patient Portal Use | 2020 | United States | Survey method | To evaluate the experiences of patients when receiving their radiology test results. |
| Gerard et al | What Patients Value About Reading Visit Notes: A Qualitative Inquiry of Patient Experiences With Their Health Information | 2017 | United States | Mixed method | To investigate the impact of OpenNotes on patients. |
| Giardina et al | Patient perceptions of receiving test results via online portals: a mixed-methods study | 2018 | United States | Mixed method | To explore the experiences of patients when viewing test results via patient portals. |
| Giardina et al | The patient portal and abnormal test results: An exploratory study of patient experiences | 2015 | United States | Qualitative method | To gain insight on how patient use patient portals to manage their care. |
| Graetz et al | Association of Mobile Patient Portal Access With Diabetes Medication Adherence and Glycemic Levels Among Adults With Diabetes | 2020 | United States | Retrospective method | To investigate the association between medication adherence and glycemic levels among adults with diabetes. |
| Graham et al | Effects of a Web-Based Patient Portal on Patient Satisfaction and Missed Appointment Rates: Survey Study | 2020 | Canada | Survey method | To determine the impact of a patient portal on patient satisfaction and health system usage. |
| Griffin et al | Patient Portals: Who uses them? What features do they use? And do they reduce hospital readmissions? | 2016 | United States | Retrospective method | To identify who uses patient portals and the impact of use/non-use of portals on 30-day hospital readmission. |
| Grossman et al | Providers' Perspectives on Sharing Health Information through Acute Care Patient Portals | 2018 | United States | Survey method | To assess the perceptions of providers about patients’ access, care delivery, and usefulness of patient portals. |
| Group Health Centre | myCARE Benefits Evaluation and Final Report | 2016 | Canada | Mixed method | To present the findings from the evaluation to capture and document implementation of a portal as part of an EHR system. |
| Grunloh et al | "Why Do They Need to Check Me?" Patient Participation Through eHealth and the Doctor-Patient Relationship: Qualitative Study | 2018 | Sweden | Qualitative method | To gain insight about the impact of personal healthcare records on the provider-patient relationship. |
| Grunloh et al | "The Record is Our Work Tool!"-Physicians' Framing of a Patient Portal in Sweden | 2016 | Sweden | Qualitative method | To understand the impact of personal health records system implementation on workflow. |
| Hanna et al | Patient perspectives on a personally controlled electronic health record used in regional Australia | 2017 | Australia | Qualitative method | To gain insight on patients’ experiences in accessing their personal healthcare records. |
| Haun et al | Large-Scale Survey Findings Inform Patients' Experiences in Using Secure Messaging to Engage in Patient-Provider Communication and Self-Care Management: A Quantitative Assessment | 2015 | United States | Survey method | To assess veteran patients’ experiences in using secure messaging. |
| Health Quality Innovation Collaborative | miDASH, Consumer Health Solution Canada Health Infoway Benefits Evaluation Health Quality Innovation Collaboration [HQIC] | 2016 | Canada | Mixed method | To present the findings from the evaluation to capture and document how implementation of a portal as part of an EHR system by focusing on quality of care, provider and patient caregiver interaction, and health service utilization. |
| Holland Bloorview | connect2care Benefits Evaluation – Results and Final Report | 2016 | Canada | Mixed method | To present the findings from the evaluation to capture and document implementation of a portal as part of an EHR system. |
| Jhamb et al | Disparities in Electronic Health Record Patient Portal Use in Nephrology Clinics | 2015 | United States | Retrospective method | To synthesize the rates of patient portal adoption and blood pressure control. |
| Johansen et al | Health Professionals' Experience with Patients Accessing Their Electronic Health Records: Results from an Online Survey | 2019 | United States | Survey method | To investigate the provider perceptions about patients accessing healthcare record. |
| Kayastha et al | Open Oncology Notes: A Qualitative Study of Oncology Patients' Experiences Reading Their Cancer Care Notes | 2018 | United States | Qualitative method | To understand the experiences of patients with advanced cancer and access to care notes. |
| King et al | Connecting Families to Their Health Record and Care Team: The Use, Utility, and Impact of a Client/Family Health Portal at a Children's Rehabilitation Hospital | 2017 | Canada | Prospective method | To examine the use and impact of connect2care portal. |
| Kummerow Broman et al | Postoperative Care Using a Secure Online Patient Portal: Changing the [Inter]Face of General Surgery | 2015 | United States | Prospective method | To evaluate the acceptance of an online care by using patient portal compared to a face-to-face consultation. |
| Leveille et al | Do Patients Who Access Clinical Information on Patient Internet Portals Have More Primary Care Visits? | 2016 | United States | Prospective method | To examine the relationship between primary care visits and access to patient portal information. |
| Lieu et al | Primary Care Physicians' Experiences With and Strategies for Managing Electronic Messages | 2019 | United States | Qualitative method | To gain insight in the primary care physicians' experiences with secure messaging. |
| Lyles et al | Refilling medications through an online patient portal: consistent improvements in adherence across racial/ethnic groups | 2016 | United States | Retrospective method | To evaluate longitudinal changes in statin adherence between racial/ethnic minorities by using the online refill function in patient portals. |
| Mafi et al | Patients learning to read their doctors' notes: the importance of reminders | 2016 | United States | Retrospective method | To determine the impact of patients accessing their providers’ notes. |
| Manard et al | Patient Portal Use and Blood Pressure Control in Newly Diagnosed Hypertension | 2016 | United States | Retrospective method | To synthesize the rates of patient portal adoption and blood pressure control. |
| Nicolas et al | The impact of a comprehensive electronic patient portal on the health service use: an interrupted time-series analysis | 2019 | Spain | Prospective method | To investigate the impact of patient portals on healthcare utilization. |
| Forster et al | Maternity patients' access to their electronic medical records: use and perspectives of a patient portal | 2015 | Australia | Retrospective method | To investigate the perceptions of maternity patients when accessing a patient portal. |
| Mendel et al | Impact of health portal enrolment with email reminders at an academic rheumatology clinic | 2017 | United States | Survey method | To explore the impact of patient portal utilization on appointment adherence. |
| Millman et al | Optimizing Adherence Through Provider and Patient Messaging | 2016 | United States | Retrospective method | To determine the effectiveness of secure messaging on healthcare. |
| Moll et al | Patients' Experiences of Accessing Their Electronic Health Records: National Patient Survey in Sweden | 2018 | Sweden | Survey method | To gain understanding about why patients access and how they use their personal health records. |
| Moll et al | Oncology health-care professionals' perceived effects of patient accessible electronic health records 6 years after launch: A survey study at a major university hospital in Sweden | 2019 | Sweden | Survey method | To examine the impact of the implementation of a personal records system on oncology health-care professionals, 6 years after launching the system. |
| Ontario Shores Centre for Mental Health Sciences | Ontario Shores’ HealthCheck Patient Portal, Ontario Shores Centre for Mental Health Sciences, Benefits Evaluation Report | 2016 | Canada | Mixed method | To present the findings from the evaluation to capture and document implementation of a portal as part of an EHR system. |
| Peremislov | Patient use of the electronic communication portal in management of type 2 diabetes | 2016 | United States | Retrospective method | To determine the effectiveness of secure messaging on healthcare for the management of type 2 diabetes. |
| Petullo et al | Effect of Electronic Messaging on Glucose Control and Hospital Admissions Among Patients with Diabetes | 2016 | United States | Retrospective method | To determine the relationship secure messaging and HbA1c levels on emergency room visits and hospital admissions. |
| Pillemer et al | Direct Release of Test Results to Patients Increases Patient Engagement and Utilization of Care | 2016 | United States | Mixed method | To investigate the impact of patients accessing test results. |
| Plate et al | Utilization of an Electronic Patient Portal Following Total Joint Arthroplasty Does Not Decrease Readmissions | 2019 | United States | Retrospective method | To assess the impact of patient portals on patients after a total hip and total knee replacement surgeries, and if secure messaging impact healthcare utilization. |
| Raghu et al | Using secure messaging to update medications list in ambulatory care setting | 2015 | United States | Retrospective method | To understand the differences between portal users and non-users, and medication responses compared between secure messaging and over phone. |
| Reed et al | Portal Use Among Patients With Chronic Conditions: Patient-reported Care Experiences | 2019 | United States | Survey method | To examine patients with chronic conditions experiences in using a patient portal |
| Reed et al | Patient-initiated e-mails to providers: associations with out-of-pocket visit costs, and impact on care-seeking and health | 2015 | United States | Survey method | To evaluate the impact of secure messaging on patient cost and healthcare utilization. |
| Reicher et al | Implementation of Certified EHR, Patient Portal, and "Direct" Messaging Technology in a Radiology Environment Enhances Communication of Radiology Results to Both Referring Physicians and Patients | 2016 | United States | Retrospective method | To evaluate the impact of secure messaging and patient engagement. |
| Rief et al | Using Health Information Technology to Foster Engagement: Patients' Experiences with an Active Patient Health Record | 2017 | United States | Qualitative method | To examine the use and impact of HealthTrak portal for patients at risk for cardiovascular disease. |
| Riippa et al | A Patient Portal With Electronic Messaging: Controlled Before-and-After Study | 2015 | Finland | Prospective method | To assess the benefits and risks of providing secure messaging options to patients with chronic conditions. |
| Robinson et al | Patient perceptions and interactions with their web portal-based laboratory results | 2019 | Canada | Qualitative method | To explore patient’s perspectives on accessing laboratory results and the impact on their health and healthcare. |
| Ronda et al | Patients' Experiences with and Attitudes towards a Diabetes Patient Web Portal | 2015 | Netherlands | Survey method | To explore patient experiences with access to patient portals. |
| Saberi et al | Antiretroviral Therapy Adherence and Use of an Electronic Shared Medical Record Among People Living with HIV | 2015 | United States | Prospective method | To examine the impact of access and no-access to patient portal on antiretroviral therapy [ART] adherence in HIV-positive individuals. |
| Shah et al | Education Research: Electronic patient portal enrollment and no-show rates within a neurology resident clinic | 2019 | United States | Retrospective method | To explore the impact of patient portal utilization on appointment adherence in a neurology clinic. |
| Shah et al | Accessing personal medical records online: a means to what ends? | 2015 | United Kingdom | Survey method | To examine the types of patients that access their healthcare records and the impact of the access on patient cost-savings. |
| Shaw et al | Increasing Health Portal Utilization in Cardiac Ambulatory Patients: A Pilot Project | 2017 | United States | Mixed method | To developed a process in engaging patients in using a portal and increase portal utilization. |
| Shimada et al | Sustained Use of Patient Portal Features and Improvements in Diabetes Physiological Measures | 2016 | United States | Retrospective method | To examine the association between prescription refills and secure messaging for the management of type 2 diabetes. |
| Sieck et al | The Rules of Engagement: Perspectives on Secure Messaging From Experienced Ambulatory Patient Portal Users | 2017 | United States | Qualitative method | To explore the experiences of patients and providers when using secure messaging. |
| Sorondo et al | Patient Portal as a Tool for Enhancing Patient Experience and Improving Quality of Care in Primary Care Practices | 2016 | United States | Prospective method | To determine the impact of portals on patients with chronic conditions and healthcare utilization and self-care. |
| Szilagyi et al | Effect of Patient Portal Reminders Sent by a Health Care System on Influenza Vaccination Rates: A Randomized Clinical Trial | 2020 | United States | Randomized Controlled trial | To examine the effectiveness of a patient portal in improving rates of influenza vaccination. |
| The Social Research and Demonstration Corporation | Impacts of direct patient access to laboratory results – Final Report | 2015 | Canada | Mixed method | To understand how direct lab access affects patients’ experience, their utilization of healthcare services, physicians’ workload and their relationships with patients. |
| Vydra et al | Diffusion and Use of Tethered Personal Health Records in Primary Care | 2015 | United States | Mixed method | To investigate the use of a patient portal by healthcare providers. |
| Walker et al | OpenNotes After 7 Years: Patient Experiences With Ongoing Access to Their Clinicians' Outpatient Visit Notes | 2019 | United States | Survey method | To investigate the perceptions of patients who access their visit notes. |
| Wang et al | Adoption of an Electronic Patient Record Sharing Pilot Project: Cross-Sectional Survey | 2020 | China | Survey method | To investigate the feasibility and acceptance of a personal health records system implementation in Hong Kong. |
| Wildenbos et al | Older adults using a patient portal: registration and experiences, one year after implementation | 2018 | Netherlands | Survey method | To examine the factors among older adults that contribute and inhibit patient portal enrollment and use. |
| Winget et al | Electronic Release of Pathology and Radiology Results to Patients: Opinions and Experiences of Oncologists | 2016 | United States | Survey method | To evaluate the experiences of patients when receiving normal and abnormal test results via a patient portal. |
| Wolff et al | Inviting patients and care partners to read doctors' notes: OpenNotes and shared access to electronic medical records | 2017 | United States | Survey method | To investigate the impact of OpenNotes on patients and caregivers. |
| Women's College Hospital | aEPR Benefits Evaluation | 2016 | Canada | Mixed method | To present the findings from the evaluation to capture and document how implementation of a portal as part of an EHR system that supports improved quality of care, patient safety and clinician efficiency. |
| Wright et al | Sharing Physician Notes Through an Electronic Portal is Associated With Improved Medication Adherence: Quasi-Experimental Study | 2015 | United States | Retrospective method | To investigate the association between medical visit notes and medication adherence in primary care. |
| Zanaboni et al | Patient Use and Experience With Online Access to Electronic Health Records in Norway: Results From an Online Survey | 2020 | Norway | Survey method | To investigate who and why uses patient portals. |
| Zhong et al | On the effect of electronic patient portal on primary care utilization and appointment adherence | 2018 | United States | Retrospective method | To explore the impact of patient portal utilization on appointment adherence. |
| Zhong et al | Characteristics of Patients Using Different Patient Portal Functions and the Impact on Primary Care Service Utilization and Appointment Adherence: Retrospective Observational Study | 2020 | United States | Retrospective method | To investigate the impact of a patient portal on secure messaging, prescription refills, and outpatient appointments. |
| Zhou et al | Personal health record use for children and health care utilization: propensity score-matched cohort analysis | 2015 | United States | Retrospective method | To examine the association between patient portal use and healthcare utilization in a pediatric settings. |
