# Supplementary material for: Capturing the Impact of Patient Portals Based on the Quadruple Aim and Benefits Evaluation Frameworks: Scoping Review
Source: J Med Internet Res. 2020 Dec 8;22(12):e24568. doi: 10.2196/24568 (PMC7755541; doi:10.2196/24568)
Supplement: Multimedia Appendix 3 [file jmir_v22i12e24568_app3.docx]

Patient perspective

| **Author/s** | **Country** | **Study design** | **Evaluated patient portal features** | **Methodological approach for evaluation** | **How was the methodology implemented** | **Study results** |
| --- | --- | --- | --- | --- | --- | --- |
| Robinson et al | Canada | Qualitative method | Viewing laboratory and diagnostic results | - Semi structured interviews | - Semi structured interviews were conducted where selective coding was used, creating higher level themes/ categories. - Questions focused on use of the portal, reception of test results and changes in healthcare experience as a result of the portal. Grounded theory analysis was used as its inductive nature makes it well suited for understanding healthcare experiences. | - Patients varied in their understanding of the results. Patients stated that healthcare providers sometimes do not provide sufficient information when commenting on results. - Benefits of access to test results included convenience, fewer appointments and decreased anxiety. Some participants described increased engagement in their healthcare and positive health changes. However, patients expressed concerns about receiving alarming test results. |
| Moll et al | Sweden | Survey method | Patient portal in general | - Survey | - An anonymous self-completion questionnaire was designed covering different portal usage topic areas with a total of 24 questions. General questions related to the portal. - There were questions targeting experiences from accessing and using the content, information security, information needs, behavior, and information-seeking style. - The questionnaire included questions with various response options [5-point Likert scale, multiple choice, and free text form]. Descriptive analysis was conducted on the responses. Reported percentages were based on those who answered each specific question. | - From the respondents, 68.41% [1737/2539] wanted access to new information same day or after a day [within 24 hours]. Additionally, 19.22% [488/2539] wanted access to new information within 2 weeks, while 1.42% [36/2539] within 1 month, and 10.95% [278/2539] chose “other.” - Respondents reported that lab results were the most important information for them to access. - No statistically significant association between respondents who were working or had been working in health care and those who had not [P=.17] in terms of availability of new results. - The study found that patients considered access to information as a means to patient empowerment and involvement. |
| Giardina et al | United States | Mixed method | Viewing laboratory and diagnostic results | - Semi structured interviews - Structured interviews | - First semi-structured interview guide was developed which was tested with patents. Once the interview guide was enhanced, the guide was finalized as a structured interview guide for the subsequent interviews. - Structured interview assessed: participant characteristics, physician and patient actions on test results, patient perceptions of receiving results via the portal, and portal concerns and suggestions for improvement. The structured interviews had 3 open-ended questions. | - From the participants, 89.5% indicated that their physician explained why the test was being ordered. Only 50.5% reported that their physician told them to check their portal for the result. - Furthermore, 63.2% of the participants reported that their physician did not include a note explaining the result. - Abnormal result [44.2%] resulted in a call than normal [15.4%] results, and 25.3% sent a secure message to their doctor regarding the test result [abnormal =32.6% and normal=19.2%]. - In addition, 61.1% of the participants saw a visual cue to know if result normal or abnormal; 16.8% reported that the physician told them it was normal or abnormal; 8.4% stated they had medical knowledge to know if test normal or abnormal; and 8.4% did not know what the results meant. - From the participants, 55.8% patients with abnormal results experienced negative emotions; 21.2% patients with normal results experienced negative emotions; 50.0% of the patients did not care and no emotions when they saw the results; and 60.0% did not have any issues. - In total, 52.6% of the participants wanted portal improvements [display, usability, and notifications]. |
| Kayastha et al | United States | Qualitative method | OpenNotes | - Semi structured interviews | - Semi structured interviews focused on how do patients with advanced cancer experience reading their own medical records. - Focused on four segments: assessing their overall experience reading notes, discussing how notes affected their cancer care experiences, having the participant read a real note with the interviewer, and, making suggestions for improvement. - Content analysis approach rooted in grounded theory was used. | - Nearly all patients described enhanced comprehension about their disease and care, because notes refreshed their memory and clarified their understanding of visits. - For a subset of patients, increased anxiety. - For others, eased uncertainty, relieved anxiety, and facilitated control. - Patients identified two areas needing improvement: the use of jargon, and repetitive information masking important updates. |
| Denneson et al | United States | Survey method | OpenNotes | - Survey - EHR administrative data | - The data was from the baseline survey of a longitudinal study examining a Web-based course designed to educate VHA patients on the use of OpenNotes. - Two items assessed participants’ ability to take ownership of their health care [health self-efficacy]. Response options range from 1, not at all, to 5, extremely. - Two items assessed participants’ sense of knowledge about their health and health care [health knowledge]. Response options range from 1, not at all, to 5, extremely. - Two items asked participants about how their relationship with their clinician had changed since reading their notes [patient-clinician alliance]. Response options range from 1, a lot less trust, to 5, a lot more trust. Or from 1, a much worse relationship, to 5, a much better relationship. - Two survey items asked participants about negative emotional responses to reading their notes [negative emotions]. Response options range from 1, never, to 5, always. | - Reading OpenNotes helped patients feel in control of their health care [49% very to extremely in control]; had a little [17%] to a lot [28%] more trust in clinicians; experienced stress or worry 26% reported sometimes experienced stress or worry and 8% reported often or always; reported feeling upset after reading their notes [18% reported sometimes felt upset, and 8% reported often or always]. - Patients with PTSD reported experiencing greater levels of negative emotional responses to OpenNotes. - Findings show small-to-null effects regarding associations with positive and negative responses to OpenNotes. - Although patients reported negative responses, but they also reported benefits in reading their notes. |
| Chimowitz et al | United States | Survey method | OpenNotes | - Survey | - The OpenNotes Safe Care Survey of patients and caregivers was conducted with the goal of measuring the impact of OpenNotes on patient and family perceptions of patient safety. - The survey focused on informal caregivers’ perceptions of the impact of reading notes on ambulatory follow-up; identification of documentation errors; and relational effects such as caregiver trust in the clinician. | - Majority of caregivers reported benefits of reading notes related to ambulatory safety behaviors. Fifty-five percent reported reading notes helped them to remember to get the patient’s tests done, and 92.3% reported reading notes helped them understand the reason for the patient’s referral to a specialist. - Among note-reading caregivers, 53.7% trusted the clinician more, and 58.2%, reported better teamwork as a result of OpenNotes. - Caregiver access to notes had little to no negative implications for caregiver-provider relationships. |
| Fossa et al | United States | Survey method | OpenNotes | - Survey - Shared decision making tool - EHR administrative data | - A survey was conducted which included questions that measured how many notes a patient read in the previous 12 months, his/her experience with clinicians, perceived risk and benefits of OpenNotes, and measures of patient satisfaction and shared decision making. - The survey included the three-item CollaboRATE scale, which measures a patient’s experience with shared-decision making. - Primary independent variable was the number of notes read by patients. - Classified patients into three categories: 1] never read a note or read a note more than 12 months ago, 2] read 1-3 notes in the past 12 months, and 3] read 4+ notes in the past 12 months. | - From the respondents, 54% had read 4+ notes, 42% read 1-3 notes, and only 4% of patients reported reading no notes. - Who read 4+ notes were 15% more likely to have top-box CollaboRATE scores for clinician effort in helping them understand health issues [p=.011]; 15% more likely for clinicians listening to the things that matter most to them [p=.009]; and 16% more likely for clinician effort in including them in the plan of care [p=.008]. - Clear correlation between what shared decision making required and the transparency OpenNotes provided. |
| Gerard et al | United States | Mixed method | OpenNotes | - Survey | - A survey was conducted to evaluate a 9-item patient feedback tool linked to OpenNotes as part of a pilot quality improvement initiative. | - From the survey respondents, 98.5% indicated that the reporting tool was valuable, and 68.8% feedback reports had qualitative responses about what patients liked about reading notes and the OpenNotes patient reporting tool process. - Patients learned about their condition through the notes. Checking accuracy was deemed as important. Patients wanted some control over the decision making. - The availability of notes made face-to-face time more effective. Patients felt positive when reading notes. Patients reported that OpenNotes and the reporting tool allowed for partnership and engagement, bidirectional communication and enhanced education, and importance of feedback. |
| King et al | Canada | Prospective method | Patient portal in general | - Survey - Focus groups - Semi-structured interviews - Patient portal administrative data | - Adopted a concurrent triangulation approach by using portal login information, a survey, and focus groups or interviews. - The survey assessed caregivers’ perceptions of the utility of and satisfaction with the portal and e-messaging, and the portal’s impact on client engagement and perceptions of caregiver-provider communication. | - Typical pattern was a steady level of use [2.5 times a month over an average of 9 months]. - A moderate degree of perceived usefulness of and satisfaction with the portal and e-messaging features, and evidence that the portal was perceived to provide useful access to the clinical record. - Some evidence that portal access facilitated caregivers’ receptions of engagement in care, but this evidence was not strong. - Little evidence that the portal led to feelings of greater involvement in the care process, improved ability to express concerns to providers or enhanced relationships with providers, or reduced number of in-person visits. |
| Rief et al | United States | Qualitative method | Patient portal in general | - Focus groups | - Focus group were conducted with participants from the active arm of an RCT. - The data was analyzed through qualitative “editing” approach by first developing a codebook and then coding the transcripts. - Goal was to capture the experience of using the active version of the PHR known as HealthTrak. | - Active reminders and tracking tools prompted both more frequent and improved communication with their providers. - Getting real time information of lab tests before appointments led to improved conversations with the provider. - Better engagement with the providers due to patient having more knowledge about their health. - The information eliminated the time pressure felt during short appointments. - Increased awareness about personal health. |
| Wolff et al | United States | Survey method | OpenNotes | - EHR administrative data - Patient portal administrative data - Survey | - EHR data was pulled for the baseline information. - At follow up, if participants viewed doctor visit notes were asked about their use and perceptions of OpenNotes. - Measures of online practices of patients and care partners were constructed from digital recordings of My Geisinger interactions for the 12-month periods before exposure to OpenNotes. | - Patients reported not viewing notes because they relied on their care partners to read the notes [18.5%], or they did not have access to a computer [16.7%]. - From the care partners, 35.5% viewed doctor notes because they were unable to attend the visit. - Shared notes increased patients’ confidence in addressing aspects of their health. |
| Wildenbos et al | Netherlands | Survey method | Patient portal in general | - Survey - Patient portal administrative data | - Registration rates for one year were collected via automated patient portal data extraction. - Patients were classified as active, declined, and expired. - An online survey in Dutch with open-ended questions collected the experiences of older adults. The survey served to gain insight in how MyChart’s functionalities could be improved. | - From the respondents, 40% found the portal useful and it allowed them to plan and follow up on upcoming appointments. Around 8% were dissatisfied with the usefulness due to incompleteness of the information and time delays for tests to show up. - Main inhibitors were that patients had higher expectations of MyChart based on their idea of what functionalities a patient portal should provide [24%]; the unresponsiveness of physicians to messages sent by patients via MyChart [15%]; and the experienced usability problems [22%]. |
| Reed et al | United States | Survey method | Patient portal in general | - Survey | - A patient experience survey was developed with guidance of patient panel with focus on examining patient health care experiences among patients with chronic conditions. | - Portal nonusers reported preferring in-person health care [54%] or experiencing internet access barriers to enrollment [41%]. Nine of 10 patients reported that it improved health care convenience, access to health information, and integrated with in-person services. - Nearly a third of users reported the portal had improved their overall health. - Many patients who did not use the portal lacked access to a connected device. - Among patients who had used the portal, 90% reported ≥ 1 aspect of convenience, 92% reported ≥1 aspect of data and information usefulness, and 92% reported that using the portal integrated with their other health care experiences. |
| Millman et al | United States | Retrospective method | Secure messaging | - EHR administrative data | - Proprietary algorithms based on evidenced-based medicine were developed for 140 treatments across 19 major diseases. - Each gap assessment algorithm used the International Classification of Diseases, Ninth Revision, Clinical Modification codes and the presence of claims [medical and pharmacy] to identify the health care services received by the member. - Based on the algorithms, gaps in care were identified. If no such test was present, a gap in care was indicated triggering a message for that individual. If a match could be made to an appropriate physician, a message was sent to her or him as well. - For each gap in care, a new regression model was created. | - Messages notifying members of gaps in care can influence evidence-based adherence for some conditions. Messages were associated with statistically significant [P < 0.05] changes in utilization related to 23 evidence-based recommendations for care. - Greatest impacts observed were for messages related to diabetes medical management considerations [HbA1c test completed] or missed therapy intervention. |
| Shimada et al | United States | Retrospective method | Secure messaging and eRefills | - EHR administrative data - Patient portal administrative data | - Used International Classification of Diseases, Ninth Revision, Clinical Modification [ICD-9-CM] diagnosis codes to determine type 2 diabetes diagnosis and determine patient characteristics at baseline. | - Of the cohort, 34.13% [38,113/111,686] was using Web-based refills, and 15.75% [17,592/111,686] was using secure messaging. - Small, statistically significant, and potentially meaningful improvement in physiological measures among diabetic patients who initiated and sustained use of Web-based refills or secure messaging or both via portal. - Rate of use and increase in use was greater for Web-based refills than for secure messaging. - Although rates of use of the refill function were higher within the population, sustained secure messaging use had a greater impact on HbA1c. |
| Reicher et al | United States | Retrospective method | Secure messaging | - Patient portal administrative data - Administrative data collected by an organization | - Focus was on determining interoperability between system and patient access to laboratory results. | - An average of 6.9% rate of use by patients who accessed their health data online, and about half of these patients were new users to the system. - Availability of radiology reports online was associated strongly with increased patient use of the system, with a likelihood ratio of 2.63. - Secure messaging allowed for efficient bidirectional radiologist- patient communication. |
| Wright et al | United States | Retrospective method | OpenNotes | - EHR administrative data - Patient portal administrative data | - To determine whether patients changed adherence status pre- and post-intervention, a four-level outcome variable was created: nonadherent to adherent, adherent to adherent, nonadherent to nonadherent, and adherent to nonadherent. | - Compared to those without access, patients invited to review notes were more adherent to antihypertensive medications [79.7% for intervention versus 75.3% for control group]. - Adherence was similar among patient groups taking antihyperlipidemic agents [77.6% for intervention versus 77.3% for control group]. - Demonstrated that patients who have access to their progress notes have a higher adherence rate to antihypertensive medications. |
| Saberi et al | United States | Prospective method | Medication | - Chart review - EHR administrative data - Medication dispensing administrative data | - The difference in refill adherence change pre- and post-portal use [for users] or before and after a randomly assigned reference date [for non-users] constituted the primary outcome measure. | - Observed stable adherence over time among portal users, compared with small declines among non-users. |
| Shah et al | United Kingdom | Survey method | Patient portal in general | - Survey | - Questionnaire comprised of five closed questions each followed by an open question. - The closed questions asked patients about access, making telephone calls, appointments due to results, and time and money saved. For each question, patients were required to answer ‘yes 'or ‘no’ and, if ‘yes’, to estimate the number of times this had been the case. - Each of the questions were followed by an open question ask-in patients to provide examples of how they had used record access. | - Greatest savings as reported by patients related to calls to the practice and appointments with doctors. - From the participants, 13% thought that they had made extra appointments with their doctor as a result of record access. - Portal savings: 27.2% reported savings in terms of time to travel, time off work, money in terms of gas and parking. - From the participants 8.7% reported better managing own health. |
| Ronda et al | Netherlands | Survey method | Patient portal in general | - Survey - EHR administrative data - Clinical outcome tools | - Survey was sent to users and non-users which contained multiple choice questions about reasons for requesting a login, the usability of portal features and patient’s wishes. Three questions that were scored on a 5-point Likert scale. - Collected patient data form the electronic health record, such as login frequency, age, gender, type of diabetes, treatment setting, laboratory values, comorbidity and diabetic complications. The patients were analyzed according to their login-frequency. - Compared two groups: patients who requested a login but never used it or only once [‘early quitters’] and patients who requested a login and used it at least two times [‘persistent users’]. - In addition to the survey, used several clinical measure tools: the Diabetes Treatment Satisfaction Questionnaire  [DTSQ]; Problem Areas in Diabetes [PAID]; Diabetes Management Self-Efficacy Scale [DMSES]; and Brief Diabetes Knowledge Test [BDKT]. | - With a higher HbA1c, the odds of becoming a persistent user decreases. - Patients wanted to enroll because they were informed by their physician. - Patients who became persistent users were apparently those with a higher disease seriousness. - Insulin use was a predictor of requesting a login. - Two thirds of the persistent users responded that they did not feel the portal supports them in most lifestyle choices. - Persistent users perceived the comprehensibility of the portal more favorably than early quitters. |
| Fiks et al | United States | Randomized Controlled Trial | Patient portal in general | - Survey - EHR administrative data | - To measure acceptability and clinical outcomes, families in both groups completed outcome surveys at enrollment [after randomization] and at 3 and 6 months. Feasibility of portal use was assessed by the proportion of participants in the intervention group who completed the portal survey each month, as a measure of whether families were able to complete the portal survey consistently. - Survey was distributed to parents. Survey results were tracked over time in a timeline available to families through the portal and to clinicians through the EHR. - Acceptability of asthma care was assessed by the 6-month outcomes survey by using 11 Likert-scaled questions developed by the study team with face validity. - Parents also completed the Parent Patient Activation Measure, Integrated Therapeutics Group Child Asthma Short Form and the Asthma Control Tool [ACT]. | - Parents of children with moderate or severe asthma used the portal more frequently [75% were frequent users compared with 47% of parents whose child had mild persistent asthma]. - Six parents felt the portal enabled them to learn more about asthma. - Value of providing decision support to families at home in addition to clinicians in the office. |
| Giardina et al | United States | Qualitative method | Viewing laboratory and diagnostic results | - Semi-structured interviews | - The semi-structured interviews included three sections: management of medical information, discussion of a specific abnormal test result, and test result notification preferences. - Interviews were analyzed through content analysis. Codes that conveyed similar meanings or ideas were combined to form new categories. | - The survey participants favored access to abnormal test results. Concerns were expressed due to the need for more timely notification and difficulty interpreting the relevance of a result. - Notification preferences appeared to be heavily influenced by past interactions with physicians and the health care system. - Patients who received an abnormal result and didn’t understand it, preferred that sensitive test results be verbally communicated by a health care professional. |
| Jhamb et al | United States | Retrospective method | Patient portal in general | - EHR administrative data - Patient portal administrative data | - Sociodemographic characteristics, comorbidities, clinical measurements, and laboratory values were captured. - The goal was to evaluate the correlation between sociodemographic, clinical factors and portal use. | - From the total number of patients, 39% access the portal and out of which 87% reviewed laboratory results, 85% reviewed medical information, 85% reviewed appointments, 77% reviewed medications, 65% requested medication refills, and 31% requested medical advice from their renal provider. - Portal adoption increased over time. - Borderline significant association between portal use and BP control [OR, 1.14; 95% CI, 1.00 to 1.29; P=0.05]. - Portal adoption was correlated with BP control in patients with hypertension; however, in the fully adjusted model this was no longer statistically significant. |
| Crouch et al | United States | Survey method | Patient portal in general | - Survey - EHR administrative data - Patient portal administrative data - Patient empowerment and satisfaction tools | - Demographics and comorbidities were collected using a self-report checklist. Patient activation was measured using the Patient Activation Measure [PAM-13]. - Patient empowerment was measured with the Health Care Empowerment Inventory [HCEI], an 8-item questionnaire with Likert scale responses used to assess the following categories: informed, engaged, committed, collaborative, and tolerant of uncertainty. - Patient satisfaction was measured with the Consumer Assessment of Healthcare Providers and Systems [CAHPS], which consists of 14 items measuring three concepts: courteous and helpful office staff, provider-patient communication, and getting timely appointments, care, and information. Medication adherence was measured using the Community Programs for Clinical Research 7-day adherence measure. | - The use of portal was associated with significantly higher levels of patient activation and levels of patient satisfaction for getting timely appointments, care, and information. - The use of My HealtheVet was associated with higher levels of activation, lower plasma HIV-1 RNA, and greater ability to correctly identify CD4 counts and viral loads. - Higher use of portal use was associated with positive clinical and behavioural characteristics. |
| Mafi et al | United States | Retrospective method | OpenNotes | - EHR administrative data - Patient portal administrative data | - Claims and online registration records to obtain demographic and clinical data, including visit dates and ICD-9-CM diagnostic codes associated with each visit. - Administrative race data was obtained by clinical registration staff. - Developed a natural experiment to assess the impact of reminders on patients’ viewing patterns. - Used information systems data from the respective patient portals to identify which notes patients accessed, when they accessed them, and how many times they accessed each note. | - In the presence of invitations and reminders to view notes, interest was high and remained durable. - At the first clinic setting viewing did not decline in year 2 even when reminders ceased. In the second setting, patients viewed notes far less frequently as soon as the reminders ceased. - Compared to white patients, black patients viewed notes less frequently [55.1% vs 36.3%, respectively, P<.001] and other/multiracial patients viewed notes less frequently [55.1% vs 50.2%, respectively, P<.001]. Race/ethnicity did not affect the persistence of notes viewed over time: black and other/multiracial subgroups continued to view notes with similar frequencies over time during the 2-year period. |
| Petullo et al | United States | Retrospective method | Secure messaging | - EHR administrative data - Patient portal administrative data | - The following data was collected: age, gender, race, most recent insurance status, most recent body mass index, diabetes type, and prior insulin use. - The primary outcome of interest was the most recent HbA1c within the observation period. | - Active secure messaging use was associated with a 0.156% lower HbA1c compared with inactive patients [P = 0.0002], and a 0.263% lower HbA1c compared with active nonusers [P < 0.0001]. - The number of messages among users, was not associated with HbA1c. |
| Graham et al | Canada | Survey method | Patient portal in general | - Survey - Patient portal administrative data | - A novel 30-question survey instrument was developed and sent to all registered portal users. - The survey focused on: satisfaction with the patient portal; utility of the patient portal; impact of the patient portal; and demographic characteristics. | - Patients had high general satisfaction, with over 90% reporting that it was easy to use, and almost half reporting that it saved them a medical visit. - From the respondents, 48% reporting avoiding a clinic visit and 2.7% avoiding an emergency department visit. |
| Garry et al | United States | Survey method | Viewing laboratory and diagnostic results | - Survey - EHR administrative data | - Survey was developed to capture the satisfaction and understanding of patients from portal usage. Developed two versions of an original 14-question survey: one form for patients who had viewed results in the online portal and another for patients who had not viewed their results on the portal. More than one choice could be selected. - Patient characteristics were pulled from the medical record. - Patient characteristics that were self-reported included race, ethnicity, education level, and health literacy. | - From total responders, 87.8% reported having received their imaging test results, with 48.4% first being notified through the patient portal, and 39.4% via direct provider communication. - One of every six patients who had MRI or CT scans [17%] reported clear understanding of their results when first receiving the results through the portal. - Satisfaction with the timing of test result notification did not differ for auto-release to patient portals compared with provider-led communication, but fewer patients understood their imaging results clearly when they first were notified through the patient portal. |
| Graetz et al | United States | Retrospective method | Medication adherence | - EHR administrative data - Patient portal administrative data | - Medication adherence was measured based on the number of days’ supply of oral diabetes prescription drug dispensed in each month of the study. - Assessed the associations of portal access with outcomes of medication adherence and HbA1c level. - Used portal administrative data to capture portal use and access device, and EHR data to capture prescription refills for oral diabetes drugs and glycemic levels, as measured by HbA1c laboratory test results, control variables, and demographic characteristics. | - For patients not previously using the portal, adding mobile access was associated with statistically significant improvements in adherence to oral diabetes drugs and lower glycemic levels. - Improvements associated with mobile portal access were greater among patients with higher clinical need at baseline [HbA1c level >8%]. - Found a more modest but still statistically significant increase in adherence among patients with lower initial glycemic levels and in the overall population estimates, translating to increased adherence of approximately 0.5 additional days per month. |
| Walker et al | United States | Survey method | OpenNotes | - Survey - Patient portal administrative data | - A survey was conducted with portal users who used portal accounts and had at least 1 visit note available in a recent 12-month period. - The main outcome measures included patient-reported behaviors and their perceptions concerning benefits versus risks. | - Patients report that reading clinical notes provided them substantial benefit. - Only a third of patients recalled discussing their notes during visits or having their clinicians recommend that they read them. - Few were very confused or more worried after reading notes. - Results strongly suggested that transparency helps patients feel more engaged in their care. |
| Devkota et al | United States | Retrospective method | Patient portal in general | - EHR administrative data - Patient portal administrative data | - Patients were offered access to a patient portal. EHR administrative data was used to capture various patient information. - Three levels of e-mail use were defined: nonusers were those not activating an account or those who activated an account but neither read nor wrote e-mails; readers activated an account and read e-mails but did not write e-mails; and readers and writers activated, read, and wrote e-mails. | - Patients who read and wrote e-mails had significantly [P<0.001] lower average HbA1c values compared to nonusers. - In adjusted analysis, patients who only read email also had significantly [P<0.05] lower mean HbA1c values compared to nonusers. - Patients with more active e-mail communication via a patient portal appeared to have the greatest likelihood of HbA1c control. |
| Manard et al | United States | Retrospective method | Patient portal in general | - EHR administrative data | - Study variables were created from International Classification of Diseases, 9th Revision, Clinical Modification [ICD-9-CM] codes. - Blood pressure measures were obtained from vital sign data available from the electronic medical record. - Sociodemographic variables available in the medical record included age, race, sex, and marital status. | - Patients who used the portal, compared with nonusers, were 24% more likely to achieve blood pressure control; however, after adjusting for sociodemographic factors, this association was no longer present. - Low rates of portal use among minorities and disadvantaged patients contributed to a decreased likelihood of achieving blood pressure control. |
| Zanaboni et al | Norway | Survey method | Patient portal in general | - Survey | - The survey was available after secure log-in on the national health portal. The survey included questions about background characteristics, use of the service, and experience with the service. Most of the questions concerning user experiences were scored on a 4-point Likert scale [1=strongly disagree, 2=disagree, 3=agree, 4=strongly agree]. - Three open ended questions for additional information. | - Patients found the service useful to look up health information [88.3%], keep track of their treatment [87.9%], prepare for a hospital appointment [64.0%], and share documents with their general practitioner [37.5%] or family [24.9%]. Most users found it easy to access their EHR online [93.1%] and did not encounter technical challenges. - From the respondents, 85.2% understood the content. - The overall satisfaction with the service was very high [92.7%]. - Clinical advantages to the patients included enhanced knowledge of their health condition [81.8%], easier control over their health status [92.6%], better self-care [87.4%], greater empowerment [73.1%], easier communication with health care providers [79.8%], and increased security [89.7%]. - Approximately one-third of all respondents thought that some documents were incomplete. |
| Forster et al | Australia | Retrospective method | Patient portal in general | - Survey - EHR administrative data | - The survey consisted of 14 closed ended questions relating to: ease of registration, identity verification, what prompted access, ease of use, value in improving patients’ abilities to understand appointments with care givers, and an overall rating of the value of the portal and the EMR. - Eight question responses were recorded on a 5-point Likert scale from strongly agree to strongly disagree. | - Majority of patients who were offered an account went on to create one. - The majority of maternity patients submitted registration forms online via the patient portal [56.7%]. - Overall, most patients were satisfied with the portal and the majority stated they would use it for future pregnancies. |
| Esch et al | United States | Mixed method | OpenNotes | - Survey - Semi-structured interviews | - Survey with free-text responses was conducted. - Conducted semi structured interviews with ‘heavy user’ patients, defined as those who read at least 8 notes in a 24-month period. | - Frequent users of OpenNotes reported positive experiences. - Patients pointed to increased trust, improved management of medications, and a stronger sense of control, and they hoped that easy access to doctors’ notes would become more widespread. - Two-thirds of the high-use patients chose not to share notes with others. |
| Reed et al | United States | Survey method | Secure messaging | - Survey | - Survey asked about the patient–provider messages for any type of condition or concern. The survey included questions about the impact of patient out-of-pocket costs. | - Patients with higher out of pocket cost-sharing for in-person visits were statistically significantly more likely to use secure messaging as their first method of contact when they had a question. - One in 3 reported that their phone contacts or office visits decreased because of secure messaging exchanges with providers. - Less than 3% reported that they would have avoided contact with their provider, and less than 1% reported that they would have instead sought care at the emergency department if they did not have access to secure messaging. |
| Haun et al | United States | Survey method | Secure messaging | - Survey - Patient literacy screening tools | - A mail-delivered paper-and-pencil survey was sent to portal users. The survey collected demographic data, assessed health literacy and eHealth literacy, and secure messaging use and perceptions. - The following tools were included in the data collection: BRIEF Health Literacy Screening Tool; Computer-Email-Web [CEW] Fluency Scale; and The eHealth Literacy Scale [eHEALS]. | - Majority of the respondents reported using secure messaging at least once a year. Less than 15% reported never using secure messaging. - Respondents reported being satisfied with secure messaging, as it provides a safe and secure communication tool that was easy to use and saves time. - A small percentage of respondents reported using secure messaging to address sensitive health topics. - Over 40% of respondents recommended that more education in how to access and use the portal was needed. |
| Raghu et al | United States | Retrospective method | Secure messaging and medication list | - EHR administrative data - Pharmacy call logs | - Demographics and patient information were obtained from the EHR. - The pharmacy call center logs phone calls made to patients in the patient record. | - Secure message response rate [49.5%] was statistically significantly lower than that for phone calls [54.8%, p<0.001]. - Time to complete medication list update was significantly higher if patients faxed the medication list [p<0.001] when compared to using secure messaging or telephone. - The time to complete difference between secure messaging and telephone was not statistically significant [p=0.41]. - Although the difference between secure messaging and phone was not significant, the authors concluded that messaging services had the potential to augment existing phone-based medication update process. |
| Dalal et al | United States | Prospective method | Care plan | - Structured interview data collection | - A modified care plan interview instrument was utilized to ask patients to identify a single Haberle recovery goal during the hospitalization. | - There was a non-significant increase in the mean concordance score for the overall care plan [adjusted p=0.13] among patient and clinician participants. - The patient portal was specifically configured to encourage patients to enter recovery goals, and this was reinforced via teach-back. |
| Broman et al | United States | Prospective method | Uploading of images and symptoms | - Survey | - A survey was conducted to determine the acceptability of an online postoperative care follow up. | - Using patent portal was effective in the postoperative care and follow up. - Seventy-six percent of patients [38 of 50] reported that they would be okay with only having an online visit for their postoperative care. |
| Wang et al | China | Survey method | Patient records | - Survey | - A survey was conducted with patient portal users. | - Patient and doctor levels of use were dependent on each other. - Among enrolled users of the portal, over 76% of patients were satisfied with its overall performance. Enrolled patients had high satisfaction levels with the portal. |
| Peremislov | United States | Retrospective method | Secure messaging | - EHR administrative data - Patient portal administrative data | - The secure messaging encounters were tallied, and the analysis proceeded to include open coding, category creation, and abstraction of themes. - Conventional content analysis was used. | - Three major themes that emerged as to why secure messages were sent: inform, instruct/ request, and question. - The portal was used for requests by patients and instruction by providers, showing relatively short message encounters with a high number of partially completed encounters, frequent lack of resolution, and a low level of involvement of diabetes specialists in secure messaging. |
| Crotty et al | United States | Retrospective method | Secure messaging | - EHR administrative data - Patient portal administrative data | - Reviewed all message sent through the patient portal. The focus was to assess for differences in the prevalence of unread messages according to sociodemographic characteristics. | - Messages sent from physicians to patients were reliably read in a timely manner. - Rate of unread messages for patients was 3.1% at 21 days. - Estimated 13% of unread messages were associated with a potential delay in care. - From all physician initiated outreach messages, 50% were unread. |
| Sorondo et al | United States | Prospective method | Patient portal in general | - Patient experience survey - Self-efficacy tool - Health state tool - EHR administrative data | - Data was obtained from the electronic medical records reports on the forms filled by participants using the portal. - Patient self-efficacy: Efficacy [CDSE] was assessed by utilizing a six-item questionnaire developed by Lorig et al. - Functional status: PROMIS Functional global items. - Patient-self-rated health state: EQ VAS using a single item EuroQol Visual Analogue Scale. - Patient experience with the primary care practice: AHRQ’s Clinician & Group Survey of Adult Primary Care 1.0 [CG-CAHPS] survey. | - The use of a patient portal among patients with chronic conditions enrolled in a care coordination program did not demonstrate a statistically significant improvement in self-efficacy, perception of health status, or patients’ experience with their primary care practice after 7 months of patient portal use. - Self-reported functional status was the only outcome measure to improve significantly. |
| Brohman et al | Canada | Mixed method | Patient portal in general | - Administrative data | - To describe the findings from a home-based remote patient monitoring system that transmitted data about a patient’s health status from home to healthcare providers through the patient use of portal. | - After many tries, the enrollment process was figured out which allowed for growth. The portal was found to be useful. Allowed family members to track the health of their loved ones. Enrollment process negatively impacted the scope of deployment. The portal was not integrated with the EMR. |
| eHealth Saskatchewan | Canada | Mixed method | Patient portal in general | - Survey - Focus group - Patient portal administrative data - EHR administrative data | - A benefits evaluation framework approach was utilized to capture and document implementation of a portal within a health setting. | - By the time of the final survey, 88% reported that the portal allowed to manage their health better. 43% of respondents indicated that they had actually shared their information with a family member or care provider by the end of the rollout. - Patients reported easy of usability: 58% of participants stated that CHIP was easy to navigate. 75% of providers did not feel that CHIP had increased their workload. By survey two, 50% of respondents felt CHIP had positively impacted their relationship with their healthcare provider. 83% of respondents confirm having access to results prior to appointment with physician results in more value. - 60% felt CHIP had resulted in an increased sense of partnership with their health care provider. 86% of respondents report a decrease in the number of visits to their health care provider. 43% indicated they had shared their personal health information with family members or care providers. Of this number, 73% report a positive impact due to sharing. Less than 1% reported using the medication reminder feature in CHIP. |
| Holland Bloorview | Canada | Mixed method | Patient portal in general | - Surveys - Semi-structured interviews | - Cross sectional survey of patients - Existing satisfaction measures - Case studies/series and focus groups - Clinician Interviews | - 97% of survey respondents [58/60] would definitely or probably recommend connect2care to other clients and families of Holland Bloorview as a tool to support care. - The average number of unique sessions per month between the months of June 2015 and October 2015was 420, ranging from 325 to as many as 473. |
| Children's Hospital of Eastern Ontario | Canada | Mixed method | Patient portal in general | - EHR administrative data | - Adopted the LEAN metholdolgy which was aligned with all indicators. - Evaluation conducted based on ‘Key Performance Indicator Workbook’ and ‘CHEOnext Strategic Directions’. | - Although it was anticipated that there would have bene more telephone calls related to results, our analysis in fact demonstrated a reduction in calls post implementation resulting in time savings of 77 minutes [1.3 hours] per month. 33% of end users avoided making a telephone call. - The average telephone calls per month increased by 17 from 48 to 65 for MyChart active users and increased by 80 from 287 to 366 for non-active users. - The majority of patients/families agreed the portal was easy to use [89%], their health information was accurate [83%], their personal information was secure and private [91%] and they felt more confident managing their health and well-being [or that of the person they care for] [70%]. - Twenty-eight percent [28%] of patients/families avoided making a telephone call to a healthcare provider because they could access health information electronically. - 90% of the participants would recommend MyChart to family or friends as a tool to support their healthcare. 205 patients/proxies have accessed MyChart over 1,800 times having over 11,000 feature hits. The overall enrollment number was less than CHEO anticipated but was on par with current literature on adoption rates. |
| Health Quality Innovation Collaborative | Canada | Mixed method | Patient portal in general | - Survey - Patient portal administrative data - EHR administrative data | - A benefits evaluation framework approach was utilized to capture and document implementation of a portal within a health setting. | - The overall responses were positive. 17.40% of responses either did not receive a prescription or don’t know if they received a prescription. 94.7% saved time by not having to travel to see the doctor. - 21.1% said they saved time by not having to arrange for childcare or other care for someone else they cared for with an average saving of 3 hours. - 100% saved money by not having to pay for gas, parking, public transit etc. 69% said they would recommend the e-Refill requests to other patients, family or friends. 63% would request all or most of their prescription refills electronically. - A significant decrease [-74%] in the wait time for a patient to interact with their health care provider. |
| Group Health Centre | Canada | Mixed method | Patient portal in general | - Survey - Focus group - Patient portal administrative data - EHR administrative data | - A benefits evaluation framework approach was utilized to capture and document implementation of a portal within a health setting. | - 99% of patients reported confidence to recommend to other. Office efficiency in terms of decreased calls [73%] and no visit necessary [48%]. - The most used functions: test results [78%]; messaging [59%]; scheduling an appointment [51%]; and prescription renewal [50%]. Ease of uses was based on easy registration [93%]; not much training needed [90%]; satisfaction with layout [90%]; and user friendly [97%]. Experienced value determined by having access to results [94%]; health information available online [94%]; messaging [90%]; prescription refills [89%]; proxy [85%]. - Use indicators [log ins, medical advice requests and medical renewal requests] showed 57,441 target uses. |
| Barrie Community Health Link | Canada | Mixed method | Patient portal in general | - Survey - Patient portal administrative data - EHR administrative data | - A benefits evaluation framework approach was utilized to capture and document implementation of a portal within a health setting. | - The patient portal acted as an “expansion of the standard 15-minute consultation appointment enabling patient access to a very valuable resource”. - From the patients’ perspective, the results were very favorable in support of a portal. Patients value the access to both their provider and their own personal health information. There was an overwhelming interest in receiving their results. - Patients demonstrated a readiness and willingness for more active engagement in the management of their health care. 27.4% of patients identified that they have used the patient portal at least once over the pilot period to request an appointment with a primary care provider. |
| Agency for Healthcare Research and Quality | United States | Mixed method | Patient portal in general | - Survey - Patient portal administrative data - EHR administrative data | - Various survey and administrative data from portal use and health records system were summarized to show impact of patient portals on decreasing disparities. | - Over 50% of the non-federal acute care hospitals in the US offer portals. Optum Institute/Harris Interactive Multi-stakeholder Health Care Environment Survey, June 2012 showed that there was a high interest in using portals and access to information. - Patients stating that they find it useful and easy. - Useful for patients due to informational supplement to verbal communication, objective indicator of health and progress in the hospital, gave patients ownership over data, and wanted access to outpatient notes as well. “If only affluent, well-educated patients can access portals and understand them, then these technologies could potentially worsen health disparities.” Disparities began with who was offered an access code. |
| The Social Research and Demonstration Corporation | Canada | Mixed method | Patient portal in general | - Survey - Semi structured interviews | - Surveys and interviews were conducted with patients to explore impact of viewing laboratory results through a patient portal. | - Service users also reported significantly higher rates of satisfaction with the overall process of having lab tests completed. Rates of lab test-related anxiety were low for both groups. Direct lab access was not associated with increased contact with physicians and their offices. - The comparison group were significantly more likely to have made contact with their health care provider while waiting for the results [28%] relative to the service users group [9%]. The odds of in-person visits were lower [OR=0.82, p < 0.1] for those who had six or more lab tests per year. - Those who first learned their results online had significantly lower odds of knowing if they needed to follow up with their physician [OR=0.37, p < 0.001]. Patients in the service users group [27%] were slightly but significantly more likely to feel some anxiety beforehand, relative to patients in the comparison group [23%]. Some anxiety was explained by an “empowerment effect” related to online access to results and related information |
| Canada Health Infoway | Canada | Survey method | Patient portal in general | - Survey | - A survey was conducted to capture the citizens’ use and interest in accessing their health information online and digitally enabled health services. | - Total market interest in selected digital health services, 2019, %: e-view personal health information 79%, e-view RX & RX history 76%, e-RX renewal request 76%, e-booking services with regular doctor 75%, appointment reminders 75%, specialist referrals tracking 74%, e-booking services with specialists & other health professionals 72%, e-view specialist referral request 69%, virtual care- secure email 64%, e-assessment of health / mental health status 64%, web & app for monitoring health/ well-being 60%, virtual care- SMS or app 59%, e-mental health tools 58%, remote patient monitoring device for home 54%, and virtual care - virtual visit 44%. Did access in past year: 2018 15%, 2019 17%. Have ever accessed 2018 17%, 2019 20%. Can currently access 2018 22%, 2019 27%. 63% were satisfied with the online access. 37% have avoided an in-person visit [doctor or ED]. 12% viewed list of current prescriptions in 2019. |
| Canada Health Infoway | Canada | Survey method | Patient portal in general | - Survey - Patient portal administrative data | - A survey was conducted to capture the citizens’ use and interest in accessing their health information online and digitally enabled health services. | - 36% of patients avoided an in-person visit. 67% of patients felt better able to manage their health. At 50% adoption, it was estimated that patients and caregivers could avoid costs estimated at $940 million in travel and expenses. |
| Canada Health Infoway | Canada | Mixed method | Patient portal in general | - Semi-structured interviews | - Conducted key informant interviews to gain insights about portal use. | - Concerns with privacy and security. The capacity to consent was incremental and situational. Most common ways of providing access was on a Case-by-Case Assessment. Establishment of proxy access for teenagers requires discussions about the capacity of a minor, cut-off age. - “Ages 0-11: A parent or legal guardian can be granted full access to MyChart. The patient will have access only with parent/guardian permission. - Ages 12-15: A parent/guardian can be granted full access to a patient’s MyChart record unless the patient advises CHEO that s/he doesn’t want that parent/guardian to have access. - Ages 16 or older: The parent/guardian will only have MyChart access if the patient gives permission by proxy.” |
| eHealth Saskatchewan | Canada | Mixed method | Patient portal in general | - Semi-structured interviews | - Conducted key informant interviews to gain insights about portal use. | - The range of technology adoption in the participant pool, spanning the full scope of the Rogers’ continuum. - Strong support for empowerment was evident and many positive views about the concept were expressed. - Patients wanted to know more and wanted access to be maintained. |
| Canada Health Infoway | Canada | Survey method | Patient portal in general | - Survey | - Survey was implemented to seek input from citizens in their ability to access health information online. | - The majority of Canadians [85%] reported they currently have a regular doctor/place of care. The majority [58%] saw multiple care providers – in addition to their regular doctor/place of care. - Approximately two-thirds of Canadians [18yrs+] were prescribed a medication in the past two years. When prescriptions were lost or damaged, most patients [83%] proactively find a way to contact the prescriber to get the medication, while 17% decide to go without the medication. - Among those who currently access medical records online, lab test results were the most common type of health information accessed – primarily via a lab testing company website. - 71% preferred to view lab results as soon as testing was complete. - Making appointments electronically was on par with results from 2016. Current levels of access to e-visit and virtual visit e-services was down significantly since 2016 [-4% e-visits and -2% virtual visits]. - Interest in these e-services had significantly increased. Among other e-services, 1 in 10 Canadians could utilize online tools for viewing and notification of specialist referrals. - Similarly, ~10% could send text [SMS] messages to consult with their doctor/ regular place of care. These e-services were of high interest to Canadians. |
| Canada Health Infoway | Canada | Qualitative method | Patient portal in general | - Semi-structured interviews | - Synthesized outcomes generated by benefits evaluations conducted at multiple sites implementing PHRs and/or eServices in Canada, across different types of care settings. | - 7%-8% Canadians reported accessing their medical records online. - 5% - 8% consulted with healthcare providers online via e-mail. - 3% - 4% visited virtually with provider online. - 10% - 12% sent an Rx renew request online. |
