# Supplementary material for: Capturing the Impact of Patient Portals Based on the Quadruple Aim and Benefits Evaluation Frameworks: Scoping Review
Source: J Med Internet Res. 2020 Dec 8;22(12):e24568. doi: 10.2196/24568 (PMC7755541; doi:10.2196/24568)
Supplement: Multimedia Appendix 4 [file jmir_v22i12e24568_app4.docx]

Population perspective

| **#** | **Country** | **Study design** | **Evaluated patient portal features** | **Methodological approach for evaluation** | **How was the methodology implemented** | **Study results** |
| --- | --- | --- | --- | --- | --- | --- |
| Hanna et al | Australia | Qualitative method | Patient portal in general | - Semi structured interviews | - Interviews lasted 30– 45 min and were audio-recorded and transcribed verbatim prior to analysis. - Interviews explored participants’ reasons for registering with and using the portals. Also, explored participants’ expectations and lived experiences of using the portal, usability, perceived effects on health outcomes, and suggestions for system improvement. | - Participants identified two advantages of portals: improved quality of care due to access to the health information; and the ability to use the information to manage their own healthcare. - Patients suggested that more providers should be using the portal. - The portal needed to be more accessible and simple to use. - Lack of awareness of portals might be one reason for low registration and use by consumers and healthcare providers. |
| Cutrona et al | United States | Randomized Controlled Trial | Reminders | - Survey - EHR administrative data | - This was a RCT where patients were assigned to one of the study arms: receipt of a portal message promoting influenza vaccines, [b] receipt of interactive voice recording call with similar content, [c] both a and b, or [d] neither [usual care]. - Tracked self-report [via portal or interactive voice response calls] of influenza vaccines administered outside the medical group. - The primary outcome was the receipt of an influenza vaccine. - Barriers were captured via a survey. | - Small but statistically significant improvement in completion of influenza vaccination among portal users receiving a portal message, an interactive voice recording call, or both. - Attained greater than 50% rates for patients to open portal messages, and over two-thirds of those who logged in during flu season opened the reminder message. |
| Lyles et al | United States | Retrospective method | Prescription request and renewal | - EHR administrative data | - Medication adherence was  calculated by the percentage of times that patients lacked a supply of medication. - Compared refill function users with those who used other online portal features but did not use the refill function. | - The most highly engaged online refill function users experienced significant improvement in statin adherence regardless of their race/ ethnicity. - Once patients started using the portal, medication adherence increased. |
| Szilagyi et al | United States | Randomized Controlled Trial | Reminders | - EHR administrative data - Patient portal administrative data | - Vaccination reminder letters were distributed through a patient portal. The letters included: information that influenza season was coming; recommendation to receive an influenza vaccine; a website link to input influenza vaccinations received elsewhere; and another website link to a containing information about influenza vaccine and video testimonials about influenza vaccination. - Patient characteristics were collected through administrative EHR data. - Metrics: opened the portal reminder letter; updated influenza vaccinations received outside the system; and clicked on the informational website link embedded in the portal letter. | - Vaccination rates were 37.5% among controls [no reminders], 38.0% in the 1-reminder group [P = .008 vs controls], 38.2% in the 2-reminder group [P = .03 vs controls], and 38.2% in the 3-reminder group [P = .02 vs controls]. - A small, statistically significant effect on increasing influenza vaccination rates among adults aged 18 to 64 years, male patients, non-Hispanic patients, and those not vaccinated in the prior 2 years. - Only 0.3% of portal user accessed the influenza educational materials. When self-reported vaccinations received elsewhere were included, influenza vaccination rates were 1.4 to 2.9 percentage points higher in the portal reminder groups. |
| Foster et al | United States | Retrospective method | Viewing laboratory and diagnostic results | - EHR administrative data - Patient portal administrative data | - Conducted a retrospective analysis of EHR patient portal records. For viewing of the diagnostic test results, an analysis was performed for both the total cohort [including those who have never activated a patient portal account] and the more limited subset with active patient portal accounts. - Analysis of the 2 populations helped address the separate impact of 2 broad barriers in patient portal usage: getting patients to activate accounts and, once activated, to utilize portal functionality such as viewing diagnostic test results. - The intent was to describe the differences between active and inactive portal status. - Epic Reporting Workbench [RWB] was used as a reporting tool within the EHR that can retrieve data based on specified query parameters related to the patient portal use. - All laboratory test and radiologic imaging orders during the emergency department encounters were captured. | - Activation rates were lower for those with only a single ED visit [7312/20,430, 35.79%] compared with either those with 2 to 3 ED visits [1770/4069, 43.50%; P<.001] or 4 or more ED visits [368/862, 42.7%; P<.001]. Overall, 8.91% of laboratory tests [18,573/ 208,635] ordered in the ED were viewed in the patient portal. - Females and white patients had higher view rates than males and non-white patients. |
| eHealth Saskatchewan | Canada | Mixed method | Patient portal in general | - Survey - Focus group - Patient portal administrative data - EHR administrative data | - A benefits evaluation framework approach was utilized to capture and document implementation of a portal within a health setting. | - By the time of the final survey, 88% reported that the portal allowed to manage their health better. 43% of respondents indicated that they had actually shared their information with a family member or care provider by the end of the rollout. - By survey two, 50% of respondents felt CHIP had positively impacted their relationship with their healthcare provider. 83% of respondents confirm having access to results prior to appointment with physician results in more value. |
| Health Quality Innovation Collaborative | Canada | Mixed method | Patient portal in general | - Survey - Patient portal administrative data - EHR administrative data | - A benefits evaluation framework approach was utilized to capture and document implementation of a portal within a health setting. | - The overall responses were positive. 17.40% of responses either did not receive a prescription or don’t know if they received a prescription. 94.7% saved time by not having to travel to see the doctor. 21.1% said they saved time by not having to arrange for childcare or other care for someone else they cared for with an average saving of 3 hours. 100% saved money by not having to pay for gas, parking, public transit etc. 69% said they would recommend the e-Refill requests to other patients, family or friends. 63% would request all or most of their prescription refills electronically. - A significant decrease [-74%] in the wait time for a patient to interact with their health care provider. |
| Group Health Centre | Canada | Mixed method | Patient portal in general | - Survey - Focus group - Patient portal administrative data - EHR administrative data | - A benefits evaluation framework approach was utilized to capture and document implementation of a portal within a health setting. | - 99% of patients reported confidence to recommend to other. Office efficiency in terms of decreased calls [73%] and no visit necessary [48%]. - The most used functions: test results [78%]; messaging [59%]; scheduling an appointment [51%]; and prescription renewal [50%]. Ease of uses was based on easy registration [93%]; not much training needed [90%]; satisfaction with layout [90%]; and user friendly [97%]. Experienced value determined by having access to results [94%]; health information available online [94%]; messaging [90%]; prescription refills [89%]; proxy [85%]. - Use indicators [logins, medical advice requests and medical renewal requests] showed 57,441 target uses. |
| Barrie Community Health Link | Canada | Mixed method | Patient portal in general | - Survey - Patient portal administrative data - EHR administrative data | - A benefits evaluation framework approach was utilized to capture and document implementation of a portal within a health setting. | - The patient portal acted as an “expansion of the standard 15 minute consultation appointment enabling patient access to a very valuable resource”. - From the patients’ perspective, the results were very favorable in support of a portal. Patients value the access to both their provider and their own personal health information. There was an overwhelming interest in receiving their results. - Patients demonstrated a readiness and willingness for more active engagement in the management of their health care. 27.4% of patients identified that they have used the patient portal at least once over the pilot period to request an appointment with a primary care provider. |
| Agency for Healthcare Research and Quality | United States | Mixed method | Patient portal in general | - Survey - Patient portal administrative data - EHR administrative data | - Various survey and administrative data from portal use and health records system were summarized to show impact of patient portals on decreasing disparities. | - Over 50% of the non-federal acute care hospitals in the US offer portals. Optum Institute/Harris Interactive Multi-stakeholder Health Care Environment Survey, June 2012 showed that there was a high interest in using portals and access to information. - Patients stating that they find it useful and easy. - Useful for patients due to informational supplement to verbal communication, objective indicator of health and progress in the hospital, gave patients ownership over data, and wanted access to outpatient notes as well. “If only affluent, well-educated patients can access portals and understand them, then these technologies could potentially worsen health disparities.” Disparities began with who was offered an access code. |
| The Social Research and Demonstration Corporation | Canada | Mixed method | Patient portal in general | - Survey - Semi structured interviews | - Surveys and interviews were conducted with patients to explore impact of viewing laboratory results through a patient portal. | - Satisfaction with the overall process for delivery of lab test results was generally high, particularly in the service users group, where 91% scored 7-10 on this item. - The comparison group were significantly more likely to have made contact with their health care provider while waiting for the results [28%] relative to the service users group [9%]. - The odds of in-person visits were lower [OR=0.82, p < 0.1] for those who had six or more lab tests per year. Those who first learned their results online had significantly lower odds of knowing if they needed to follow up with their physician [OR=0.37, p < 0.001]. - Patients in the service users group [27%] were slightly but significantly more likely to feel some anxiety beforehand, relative to patients in the comparison group [23%]. Some anxiety was explained by an “empowerment effect” related to online access to results and related information |
| Canada Health Infoway | Canada | Survey method | Patient portal in general | - Survey | - Survey was implemented to seek input from citizens in their ability to access health information online. | - The majority of Canadians [85%] report they currently have a regular doctor/place of care. The majority [58%] also see multiple care providers – in addition to their regular doctor/place of care. - Approximately two-thirds of Canadians [18yrs+] were prescribed a medication in the past two years. When prescriptions were lost or damaged, most patients [83%] proactively find a way to contact the prescriber to get the medication, while 17% decide to go without the medication. - Among those who currently access medical records online, lab test results were the most common type of health information accessed – primarily via a lab testing company website. - 71% preferred to view lab results as soon as testing was complete. - Making appointments electronically was on par with results from 2016. Current levels of access to e-visit and virtual visit e-were down significantly since 2016 [-4% e-visits and -2% virtual visits]. - Interest in these e-services had significantly increased. Among other e-services, 1 in 10 Canadians could utilize online tools for viewing and notification of specialist referrals. - Similarly, ~10% could send text [SMS] messages to consult with their doctor/ regular place of care. These e-services were of high interest to Canadians. |
| Canada Health Infoway | Canada | Survey method | Patient portal in general | - Survey | - Survey was implemented to seek input from citizens in their ability to access health information online. | - Many PHR initiatives in Canada. More knowledge of their health increased [78- 94 %]. More confident managing their health [78- 95 %]. - More involved in their health care 77%. Allowed them to have more informed discussions with their doctor 93 %. |
