# Supplementary material for: Capturing the Impact of Patient Portals Based on the Quadruple Aim and Benefits Evaluation Frameworks: Scoping Review
Source: J Med Internet Res. 2020 Dec 8;22(12):e24568. doi: 10.2196/24568 (PMC7755541; doi:10.2196/24568)
Supplement: Multimedia Appendix 5 [file jmir_v22i12e24568_app5.docx]

Table 4: Healthcare workforce perspective

| **Author/s** | **Country** | **Study design/ characteristics** | **Evaluated patient portal features** | **Methodological approach for evaluation** | **How was the methodology implemented?** | **Study results** |
| --- | --- | --- | --- | --- | --- | --- |
| Grünloh et al | Sweden | Qualitative method | Patient portal in general | - Semi-structured interviews | - The main research question was: “how do physicians view the idea of patient participation in general and in particular in relation to patient accessible electronic health records [PAEHR]?” - Interviews were transcribed, translated, and repeatedly read by all authors. Researchers used the same template for questions to cover the required areas of interest. Complete coding was used for the thematic analysis. | - Providers not necessarily opposed to patients having access. But providers needed to understand the needs of the patient and then guide the patient toward the right treatment. - Providers stated that portals have negative impact on "the anxious, the layperson, the child, the detail-focused, the overwhelmed, and the shopper patient". - “Patient empowerment” as a problematic concept. |
| Bush et al | United States | Mixed method | Patient portal in general | - Survey - Semi-structured interviews | - A survey was distributed among healthcare providers. - The first part of the survey captured areas such as quantified demographics; attitude toward technology adoptions [respondent’s type and frequency of social medial use]; and number of e-mails, telephone calls, and secure messages received monthly from patients to approximate practice volume. - The second consisted of 15 questions with a 5-item Likert-type scale ranging from strongly disagree to strongly agree to capture responders’ perceptions of the effect of the portal on workload, telephone calls, patient satisfaction, number of patient visits, patient quality of care, treatment compliance, professional satisfaction, and impact on clinical income. | - From the survey respondents, 72% were neutral as to whether it was easy to enroll patients, while 60% of the respondents stated that it improved patient care; 52% stated that it improved patient adherence. - Majority stated that the portal implementation did not negative impact on their salary but 43% believed that the portal increased their workload. |
| Cajander et al | Sweden | Qualitative method | Patient portal in general | - Semi-structured interviews | - Semi-structured interviews were conducted with nurses to determine their views of how the portal implementation had impacted their work. - The interviews were transcribed and complete coding was used for the thematic analysis. | - Due to access to the medical record, patients came pre-prepared with more informed questions which led to “more in-depth discussions that took time for the nurses.” - Nurses were stressed over the fact that now patients could read the notes with the potential to read about serious diagnoses. Increased transparency requires providers to consider how they chart and what words were being used. Increased workload due to duplication on services. - Patients would send an email and call over the same questions. Nurses had to respond through both communication pathways. Nurses did not have the appropriate knowledge to educate/train patients. Nurses acknowledged that patients were more informed which lead to better provider-patient relationship. |
| Johansen et al | United States | Survey method | Patient portal in general | - Survey | - A survey was developed which was based on a questionnaire used during a pilot study that preceded the study pilot study was used as a template for the survey development. - The questionnaire was pilot-tested by four researchers several times until no suggestion for modification came up. No questionnaires were excluded from the analysis due to incomplete answers. | - 25.6% of administrative staff had received feedback from patients and/or their relatives regarding mistakes or missing information in their health record. 36.4% clinical and 36.8% administrative staff had received questions from the patients and/or their relatives related to use of the PAEHR. - More doctors than nurses claimed they changed their way of reporting, and twice as many doctors than nurses worried that they will have to spend more time reassuring patients, or their relatives, after they read their record. |
| Grossman et al | United States | Survey method | Patient portal in general | - Survey | - A healthcare provider survey instrument was developed. The instrument was based on provider surveys from previous studies as well as the Telemedicine Satisfaction and Usefulness Questionnaire. - The instrument was tested and reviewed by the study team, which included 3 clinicians and an expert in questionnaire development. Questions used both negatively and positively worded stems to guard against acquiescence. - The final survey contained 5 items on inpatients' technology use, 8 items on perceptions of the portal and its impact on care, and 8 items on usefulness of portal features | - Providers did not see their patients as technology users. Perceiving certain patients as less likely to use technology impacted how providers promoted acute care portals to patients. - Every provider reported that displaying laboratory test results in the portal was moderately useful or very useful. |
| Moll et al | Sweden | Survey method | Patient portal in general | - Survey | - A questionnaire was developed which contained 12 multiple-choice questions covering the following areas: demography [age and years of working in health care]; perceived effects on contact with patients; perceived effects on documentation practices; perceived effects on quality of care; attitude towards the portal system [Journalen]; and effects supposedly experienced by patients. Four questions had binary answers [yes/no] and the rest had either interval choices [2 demographic questions] or 4-point Likert-type scale choices [7 questions]. | - Results mainly showed moderate effects in the different areas that were surveyed. In most cases, no statistically significant differences between physicians and nurses could be found. - When asked if it was generally a good idea to make medical records available through the portal, 73% of the physicians and 79% of the nurses agreed or partly agreed. The results also showed that the majority of both physicians and nurses believe that the medical notes were confusing for most patients and that patient’s felt more worried after accessing their records. |
| Vydra et al | United States | Mixed method | Patient portal in general | - Focus group - Survey | - A focus group was conducted with providers. An audio recording of the focus group was transcribed using detailed transcription, by focusing not only verbal content but also on the conversational features such as pauses, stuttering, and interruptions. This allowed for the capture of emotions, such as enthusiasm and discomfort, in addition to content. - Electronic survey was sent out to all primary care physicians affiliated with the medical center that were part of the MyChart implementation. The surveys asked physicians to estimate the average amount of time spent per week on MyChart activities, the average number of e-mails received by patients through MyChart, the average number of new activations of patient accounts, and the average number of prescription requests. | - Among the factors contributing to patient-level adoption of PHRs, aggressive marketing by providers was identified as the strongest factor. - Physicians estimated spending an average of 12.5 hours per week logged into MyChart; however, institutional records indicate an average of 8.2 hours per week [p = 0.034]. Lower adoption rates of PHRs among older physicians. |
| Grünloh et al | Sweden | Qualitative method | Access to medical records and log list | - Semi-structured interviews | - Semi structured interviews were conducted with physicians from different specialties. Interviews were transcribed and translated. A theoretically informed thematic analysis was performed. | - The thematic analysis revealed 4 main themes: work tool, process, workload, and control. - Physicians saw themselves as owners of the records. The transformation of the medical record to a patient portal was seen as time consuming and a threat to the effectiveness of their work tool. - Paper access to record was not seen as controversial, but portal access was. The physicians described that patients who demand for them to sign various forms or ask for explanations at once would interfere with their work processes. |
| Winget et al | United States | Survey method | Viewing laboratory and diagnostic results | - Survey | - Developed a questionnaire to obtain the opinions and experiences of practicing oncologists approximately 4 months after the portal was implemented. - The questionnaire included six Likert-scale questions: four about oncologist opinions and two about their experiences with release of radiology and pathology results to patients, and one open-ended additional comment field. | - Oncologists agreed that patient online access to abnormal radiology/ pathology results had negative consequences [87%], whereas opinions were more mixed for normal results. - A strong majority of respondents wanted, at most, 14 days to communicate results to patients, only 27% wanted 7 days [the current embargo]. |
| Lieu et al | United States | Qualitative method | Secure messaging | - Semi-structured interviews | - Conducted semi structured, in-depth telephone interviews with primary care physicians, including internists and family practice physicians, and primary care chiefs. The questions focused on identifying local practices with a range of group-level strategies for inbox support. | - Participants expressed mixed feelings about the increased ease of patient access created by secure portal messaging. All participants described electronic messaging as having led to increased work outside normal work hours. - Several participants reported anxiety from the lack of limits on the volume of electronic messages. Physicians described varying approaches to timing their replies to patient messages. |
| Åkerstedt et al | Sweden | Survey method | Patient portal in general | - Survey | - A survey was developed based on the questionnaire “Questions on violence and threats about violence”. Two variations of the same basic questionnaire were constructed. At the very end of the questionnaire, the respondents were given a chance to give their overall comments. | - Two out of five of respondents indicated that they believed that risks of threats and violence increased as patients gain access to their online EHRs. - The psychiatric staff were more prone than the emergency staff to believe that the risks would increase. These differences between the groups were, however, not statistically significant. |
| Shaw et al | United States | Mixed method | Patient portal in general | - Survey | - A survey was conducted with the two nurse navigators who were part of an educational intervention for post-operative patients. | - Nurses reported barriers to providing portal education due to inconsistent patient volumes, low referrals, understanding, and patients had subsequent appointments. |
| Federman et al | United States | Qualitative method | After visit summary / clinical summary | - Focus groups - Semi-structured interviews | - Focus groups and interviews were conducted with clinicians in adult primary care practices serving socioeconomically diverse communities. Focus group and interview transcripts were coded and analyzed following standard qualitative methods. | - Core themes included the use and purpose of the AVS, content modification and prioritization, formatting improvements, customization, privacy and accuracy concerns, and clinician workflow concerns. Clinicians viewed the AVS as a valuable tool for communicating health care information. - They emphasized the need for improvement. There was dissatisfaction with the EHR-generated AVS. Clinicians raised concerns about the risk of violating patient privacy and challenges to clinician workflow. |
| Sieck et al | United States | Qualitative method | Secure messaging | - Semi-structured interviews | - All interviews were conducted by telephone by using a semi structured interview guide. The interviews were transcribed verbatim to permit rigorous qualitative analysis by focusing on the themes involving secure messaging. | - Providers appreciated the ability to use secure messaging for communication. An electronic record of exchanges as a benefit. Concerns about overuse and security of information expressed by providers in pre-implementation studies may no longer apply as users gain experience. - Providers articulated a lack of clarity as to the appropriate way to communicate via MyChart and suggested that additional training for both patients and providers might be important. |
| Pillemer et al | United States | Mixed method | Viewing laboratory and diagnostic results | - Semi-structured interviews | - Conducted qualitative interviews with physicians. The interviews were conducted via telephone by the study authors and were audio recorded and transcribed. - The physicians were part of an EHR advisory panel of practicing primary care physicians. | - The interviewed physicians were concerned about patient anxiety resulting from patient portal test release. Several providers described experiences in which patients contacted them for abnormalities that were clinically insignificant, increasing the clinician’s workload. Some physicians perceived that quick interpretations of the results eliminated patient anxiety. |
| Broman et al | United States | Prospective method | Uploading of images and symptoms | - Survey | - A survey was conducted with surgeons to compare their views about the efficacy of online vs in-person visits, amount of time required for patients and surgeons to complete each visit type, and agreement between findings on online vs in-person evaluations. | - Using patent portal was effective in the postoperative care and follow up. - Surgeons reported that online and clinic visits were equally effective for 68% [34 of 50] of patients; the clinic visit was more effective for 24% [12 of 50] and the online visit was more effective for 8% [4 of 50]. - Evaluative findings for online vs clinic visits were generally in agreement. |
| Wang et al | China | Survey method | Patient records | - Survey | - A self-administered surveys were sent by postal mail to registered doctors. The survey contained questions on subjects’ awareness, acceptance, and perceptions of portals, perceived benefits and obstacles of participating in the program, reasons for not using the system after enrolling, and perceived areas for service improvement of the system. | - Patient and doctor levels of use were dependent on each other. Among enrolled users of the portal, 67% of doctors were satisfied with its overall performance. Enrolled patients, in general, had higher satisfaction levels than enrolled doctors [P < 0.001]. Around 10% [[40+10]/409, 12.2%] of enrolled doctors were dissatisfied or very dissatisfied with the online system, which was far higher than that among enrolled patients [[1+1]/501, 0.4%] |
| King et al[55] | Canada | Prospective method | Patient portal in general | - Survey - Patient portal administrative data - Semi-structured interview | - Adopted a concurrent triangulation approach, in which quantitative and qualitative data were collected at the same time and integrated at the level of interpretation. | - Service providers were concerned about how to best manage their investment of time and effort [the use, effort, and investment in the portal theme]. |
| Women's College Hospital | Canada | Mixed method | Patient portal in general | - Survey - EHR administrative data | - Surveys were distributed to each wave cohort of healthcare providers to capture their experiences with portal implementation. | - The implementation was done in 3 wave cohorts of providers and locations. Wave 2 respondents exhibited statistically significant rates of dissatisfaction with respect to training and support as compared to the Wave 1 and Wave 3 cohorts. - The Physician cohort exhibited statistically significant rates of dissatisfaction with respect to completion of clinical documentation using Epic than did their Nursing and Health Discipline cohorts. Issues were reported with the scheduling, workflow, technology, incomplete information, and inappropriate work templates. Highest satisfaction was reported with access to service. |
| Barrie Community Health Link | Canada | Mixed method | Patient portal in general | - Survey - Patient portal administrative data - EHR administrative data | - A benefits evaluation framework approach was utilized to capture and document implementation of a portal within a health setting. | - 100% of the champion users of the portal indicated that the portal had improved the communication and interactions between themselves and their patients. - 52.3% of providers believed that the portal had not improved their interactions with patients. 73.7% of patients rated that the portal improves transparency in the care that they receive. |
| Canada Health Infoway | Canada | Mixed method |  | - Semi-structured interviews - Environmental scan | - To gain understanding about what information patients were sharing [or want to share] with their health care providers that would contribute to providers’ understanding of who they were as people, beyond who they were as patients | - "Getting to Know Me" forms have been developed in various health care systems to assist providers to learn about their patients. These type of documents go under various names such "All About Me", "Patient Story", "We Ask Because We Care", "Know Me", "Patient Preference Passport". - In an evaluation done on Phase 1, the majority of staff [91%] reported that the form was easy to use, with 70% reporting it was not time consuming, 74% citing that it decreased patient agitation and distress, 61% reporting that it led to a decrease in the use of physical or chemical restraints, and 79% reporting increased satisfaction with work. |
