# Supplementary material for: Capturing the Impact of Patient Portals Based on the Quadruple Aim and Benefits Evaluation Frameworks: Scoping Review
Source: J Med Internet Res. 2020 Dec 8;22(12):e24568. doi: 10.2196/24568 (PMC7755541; doi:10.2196/24568)
Supplement: Multimedia Appendix 6 [file jmir_v22i12e24568_app6.docx]

Health system perspective

| **Author/s** | **Country** | **Study design/ characteristics** | **Evaluated patient portal features** | **Methodological approach for evaluation** | **How was the methodology implemented?** | **Study results** |
| --- | --- | --- | --- | --- | --- | --- |
| Nicolas et al | Spain | Prospective method | Patient portal in general | - EHR administrative data | - The electronic health record data were used in the analysis. Pre-period observations and trends acted as controls for post-period observations. - Outcomes were any hospitalization, any emergency department use, any 30-day all-cause readmission and number of outpatient visits. | - The rate of hospital admissions decreased by 0.44/10 000 per month [5.28/10 000 per year] in the post-intervention period. The rate of 30-day all-cause readmission decreased by 0.44/10 000 per month [5.2/10 000 per year] after the implementation of the portal. - The portal was not associated with an increase in outpatient visit rates. The portal led to no changes in service use for persons with a malignant hematological disease. |
| Leveille et al | United States | Prospective method | OpenNotes | - EHR administrative data - Ambulatory care claims data | - From the EHR data repository, information about dates of hospital admissions and emergency department visits that occurred during the study period was obtained. - Obtained patient-level diagnoses from ambulatory care claims data. - Examined patient characteristics and health care utilization according to baseline portal login frequency within each site. - Login days were categorized according to the average number of login days per month: no login days, fewer than 2 login days per month, and 2 or more login days per month. - The goal was to examine the relationship between portal use and primary care visits. | - In the first 2 months of the 2-year period, 14% of 44,951 primary care patients engaged in portal use 2 or more days per month, 31% did so 1 day per month, and the remainder had no portal use. Less than 0.1% of patients engaged in high levels of portal use. Office visits led to subsequent clinical portal use. - Did not observe an increase in the correlation between login days and primary care visits. - Could not confirm specific relationships between patients viewing their records and, as a result, choosing to make appointments to see their primary care providers. |
| Dexter et al | United States | Retrospective method | Secure messaging | - EHR administrative data - Patient portal administrative data | - A retrospective time series analysis of the correlation between the rate of MyChart messages and incoming telephone calls. - Consecutive monthly differences for both MyChart messages per 1000 patients and telephone calls per 1000 patients were calculated. - Portal use was defined as: electronic patient-to-physician messaging. - Incoming calls were defined: calls placed during regular business hours to the clinic. - Panel size was defined: the number of unique patients who have had a visit with their primary care provider at the provider’s clinic within the past 3 years. | - The hypothesis was that increase in secure messages would decrease telephone calls. - Four clinics were part of the study. Electronic patient-to-provider messaging was significantly positively correlated with incoming telephone calls at 2 of the 4 clinics [*r=*0.546, *P* < .001 and *r* = 0.543, *P* < .001]. For the other 2 clinics, there was no indication of even a slight decrease in telephone calls. - As secure messages increased, so did the telephone calls. Staff had to adjust workflow to handle both approaches. Patients did not know how long to wait for an answer through the portal, so additional calls were made. - Assumption was made that appointment scheduling increased the number of calls. Several issues with the appointment system were identified. |
| Bryan et al | United States | Retrospective method | Secure messaging | - EHR administrative data | - This was a retrospective cohort study used EHR administrative data. - Focus was on capturing the association between the number of messages sent and the number of traditional encounters. Examine the differences in number of traditional encounters and patients who sent at least 1 message the year after activation and those who sent none. | - 28% patients sent at least 1 message. Patients who sent messages were more likely to be female [63.9% vs 58.0%, P<0.001], white [92.2% vs 90.0%, P< 0.001], and have depression [27.0% vs 24.2%, P <0.001] than those who sent none. - Patients who sent messages had a greater number of traditional encounters the year after activation than those who sent none [mean 17.6 vs 11.4, P< 0.001]. Also had both more in-person office visits [7.6 vs 5.0, P< 0.001] and telephone calls [9.9 vs 6.4, P< 0.001]. - As the number of messages sent increased, so did the number of encounters. Patients who sent 1 message had 2.4 times greater odds of having a traditional encounter. |
| Zhou et al | United States | Retrospective method | Patient portal in general | - EHR administrative data - Patient portal administrative data | - Portal administrative data was used to evaluate the levels of PHR use by pediatric patients and their caregivers. - The intent was to evaluate the associations between PHR use and health care utilization. - PHR use was defined as the total number of features accessed on behalf of the child by the child’s caregiver. - Calculated the average number of outpatient clinic visits, telephone encounters, and emergency department visits between PHR-registered and nonregistered children. The data was from when children were 18 months of age until they reached 2.5 years or until they became disenrolled as members. - Message thread was defined as: the initial secure message along with all responses either by a proxy or the health care provider. - Counted each secure message thread once. | - PHR users had a higher mean number of ambulatory care visits [5.2 vs 4.1; P<.0001] and telephone encounters [3.5 vs 2.6; P<.0001]. - The difference in the number of emergency department visits was not statistically significant between users and non-users. - PHR use lead to the identification of additional health concerns that lead, in turn, to increased use of outpatient clinic visits and telephone encounters. - Occasional PHR use was not associated with higher health care utilization. - PHR use by caregivers was associated with statistically significant increases in outpatient clinic visits and telephone encounters among pediatric patients. |
| Riippa et al | Finland | Prospective method | Secure messaging | - EHR administrative data | - The cost of primary health care contacts during the 6 months before the intervention and in the 6 months following were calculated for each individual, as were the costs of providing access to the portal. - The use of primary health care resources was collected directly from the patient administration system. - Extracting the patient-level was done by using the Ambulatory and Primary Care Related Patient Groups [APR] grouper software. | - Costs decreased by an average of €91 in the unadjusted model, but increased by €48 in the adjusted model. - The unadjusted analysis showed an 89% probability of cost-effectiveness with no willingness to pay for increased patient activation, whereas in the adjusted sample, the probability of the portal being more cost-effective than care as usual exceeded 50% probability at a willingness to pay €700 per clinically significant increase in patient activation score. - Although no statistically significant improvement [>90% probability] in cost-effectiveness was detected, the results indicated over 50% probability for cost-effectiveness of the intervention at a willingness to pay €18 per 1-point increase in the patient activation score. - The results of the cost-effectiveness analysis show some support for the cost-effectiveness of a simple electronic patient portal that provided patients access to their own health records and secure messaging with the health care provider. |
| Zhong et al | United States | Retrospective method | Patient portal in general | - EHR administrative data - Patient portal administrative data | - Difference-in-Difference model for heterogeneous enrollment and treatment times was developed. - The intent was to investigate the use of primary care services by patients, adjusting for their disease burden and allowing for time-dependent portal effect. - The main outcome measures were disease burden adjusted rates of office visits arrived, no-show, and cancellation to primary care physicians per quarter between patient portal users and non-users. | - The quarterly primary care physicians’ office visit rate ratio [RR] of patient portal users to non-users was 1.33 [95% CI, 1.27–1.39; p < 0.001]. - The quarterly no-show rates of the users were significantly smaller except for the seventh, eighth and tenth quarters post adoption. Quarterly cancellation rates were not significantly affected by portal adoption [p >0.05 for all cases]. - Portal users were found to have fewer office visits. No-show rate of the user group were significantly lower than that of non-users. - The differences in cancellation rate was not significant between users and non-users. |
| Zhong et al | United States | Retrospective method | Patient portal in general | - EHR administrative data | - Demographics and patient information were obtained from the EHR, in addition to their active problem number [APN]. - Patient’s APN was considered as a time-varying confounder to account for individual disease burdens. - To characterize portal usage patterns, focused on four major portal functions: messaging, laboratory, medication, and appointment. - The intent was to evaluate how portal usage affects primary care service utilization and appointment adherence. - Patients’ office visits and telephone encounters were used as an indicator of the overall primary care service utilization. | - A high propensity to adopt patient portals did not necessarily imply more frequent use of portals. - The number of active health problems was significantly negatively associated with portal adoption [all *P*<.001] but was positively associated with portal usage [all *P*≤.01]. The same was true for being enrolled in Medicare for portal adoption and message usage. - The no-show rates were significantly lower in most quarters postadoption. Users were lower by 30% on average than nonusers. - Patients using more messaging and messaging and laboratory combined had a larger reduction in no-show rates [P<.001]. |
| Sorondo et al | United States | Prospective method | Patient portal in general | - EHR administrative data | - Data was obtained from the electronic medical records reports on the forms filled by participants using the portal. | - Emergency department visits/ 1000 patients were reduced by 26% and 21% in the intent to treat and users groups, respectively. - Hospital admissions/ 1000 patients were reduced by 46% in the intent to treat group and by 38% in the users group. |
| Mendel et al | United States | Survey method | Appointment reminders | - EHR administrative data - Patient portal administrative data | - Baseline measures were captured from the administrative data. - No-shows were studied through a prospective chart review and telephone interviews. - Process measures: portal enrolment, email reminder receipt, and call volumes. - Outcome measures were no-shows and patient and staff satisfaction. | - Reminders were associated with higher patient satisfaction and decrease in no-show appointments after 3.5 months, but it was not sustained thereafter. - Promoting portal for reminders, increased immediate enrollment. - Technical issues and lack of reminders did not decrease no-show appointments. |
| Plate et al | United States | Retrospective method | Secure messaging | - EHR administrative data - Patient portal administrative data | - Usage and healthcare utilization data were analyzed. | - Active MyChart status was not associated with 90-day ED return. For patients who sent 2 or more MyChart messages, a provider or staff response rate of <75% was significantly associated with 90-day readmission [P=.004] and showed greater 90-day ED visits that neared statistical significance [P=.070]. |
| Dumitrascu et al | United States | Retrospective method | Patient portal in general | - EHR administrative data | - EHR was used to obtain: patient demographics, clinical information, and hospital outcomes [30-day readmission, 30-day mortality, and inpatient mortality]. | - Out of the admitted patients with a portal account, 20.8% accessed the portal while in the hospital. - Compared to patients who did not access the portal, patients who accessed the portal had fewer elective admissions [54.2% vs 64.1%], were more frequently admitted to medical services [45.8% vs 35.2%], and were more likely to have liver disease [21.9% vs 12.9%] and higher disease severity scores [0.653 vs 0.456]. - There was no statistically significant difference between the users and non-user cohorts. |
| Shah et al | United States | Retrospective method | Appointment reminders | - EHR administrative data - Patient portal administrative data | - A retrospective chart review of: date of scheduled clinic visit, appointment status, MyChart enrollment, sex, age, race, preferred language, and method of referral to the clinic. Patients were defined as [1] no-shows, [2] showed, and [3] canceled. | - Portal users had lower rates of no-show appointment compared to non-users. - Rate of cancellation was similar between users and non-users. |
| Griffin et al | United States | Retrospective method | Patient portal in general | - EHR administrative data - Patient portal administrative data | - Compared the average number of times active and light users used specific features in the 30 days after discharge. | - Approximately 15% of patients were readmitted within 30 days; 15% were non-users, 13% were light users, and 21% were active users. Only 16% used the patient portal within 30 days of discharge. - Statistically significant difference [p<0.05] in 30-day readmission between non-users and active users. For patients who were active users, the odds of being readmitted within 30 days was 66% higher than patients who were non-users. |
| Graham et al | Canada | Survey method | Patient portal in general | - Patient portal administrative data - EHR administrative data | - No-show rates were calculated for users with an active patient portal account at the time of the visit and compared to rates for users attending the same clinics without a patient portal account at the time of the visit. | - 53% relative reduction in the no-show rate seen in patient portal users in the 5 pilot clinics. |
| eHealth Saskatchewan | Canada | Mixed method | Patient portal in general | - Patient portal administrative data - EHR administrative data | - A benefits evaluation framework approach was utilized to capture and document implementation of a portal within a health setting. | - The patient portal data showed decrease in face-to-face visits. |
| Ontario Shores Centre for Mental Health Sciences | Canada | Mixed method | Patient portal in general | - Patient portal administrative data - EHR administrative data | - A benefits evaluation framework approach was utilized to capture and document implementation of a portal within a health setting. | - The percentage of missed appointments was calculated by each participating clinic, and a comparison of the percentage change was made. - It showed that portal users missed 18% of total appointments compared to non-portal users' 20% missed appointments for 2015. It showed that portal users missed 9% of total appointments compared to non-portal users' 14% missed appointments for 2015. |
| Canada Health Infoway | Canada | Survey method | Patient portal in general | - Survey | - A benefits evaluation framework approach was utilized to capture and document implementation of a portal within a health setting. | - Making appointments electronically was on par with results from 2016. Current levels of access to e-visit and virtual visit e-services was down significantly since 2016 [-4% e-visits and -2% virtual visits]. Interest in these e-services had significantly increased. - Among other e-services, 1 in 10 Canadians can currently utilize online tools for viewing and notification of specialist referrals. Similarly, ~10% can currently send text [SMS] messages to consult with their doctor/ regular place of care. These e-services were of high interest to Canadians. |
