# Supplementary material for: Capturing the Impact of Patient Portals Based on the Quadruple Aim and Benefits Evaluation Frameworks: Scoping Review
Source: J Med Internet Res. 2020 Dec 8;22(12):e24568. doi: 10.2196/24568 (PMC7755541; doi:10.2196/24568)
Supplement: Multimedia Appendix 7 [file jmir_v22i12e24568_app7.docx]

Table 6: Mapping the peer-reviewed studies according to the Benefits Evaluation Framework

| **Study** | **System Quality** | | | **Information Quality** | | **Service Quality** | **Care Quality** | | | **Access** | |
| --- | --- | --- | --- | --- | --- | --- | --- | --- | --- | --- | --- |
|  | Functionality | Performance | Security | Content | Availability | Responsiveness | Patient Safety | Appropriateness/ effectiveness | Health outcomes | Ability of patients/providers to access services | Patient and caregiver participation |
| Shaw 2017 | X | X |  | X |  |  |  | X |  | X |  |
| Gerard 2017 |  |  |  | X | X |  |  |  |  | X | X |
| Giardina 2018 |  |  |  | X | X |  |  |  |  | X |  |
| Dumitrascu 2018 |  |  |  |  |  |  |  |  | X | X |  |
| Grunloh 2018 |  |  |  |  |  |  |  | X |  | X |  |
| Kayastha 2018 |  |  |  | X | X |  |  |  |  | X | X |
| Bush 2017 |  |  |  |  |  |  |  |  |  |  |  |
| King 2017 |  |  |  | X | X |  |  |  |  | X | X |
| Denneson 2018 |  |  |  |  |  |  |  |  |  | X |  |
| Chimowitz 2018 |  |  |  |  |  |  |  |  |  | X | X |
| Cajander 2018 |  |  |  |  |  |  |  |  |  |  |  |
| Zhong 2018 |  |  |  |  |  |  |  |  |  | X |  |
| Moll 2018 |  |  |  | X | X |  |  |  |  | X | X |
| Plate 2019 |  |  |  |  |  |  |  |  |  | X |  |
| Shah 2019 |  |  |  |  |  |  |  |  |  |  |  |
| Robinson 2019 |  | X |  | X | X |  |  |  |  | X |  |
| Fossa 2018 |  |  |  |  |  |  |  |  |  |  |  |
| Reed 2019 |  |  |  | X | X |  |  |  |  | X | X |
| Cutrona 2018 |  |  |  |  |  |  |  | X |  | X |  |
| Walker 2019 |  |  |  |  |  |  |  |  |  | X | X |
| Foster 2019 |  |  |  |  | X |  |  |  |  | X |  |
| Dalal 2019 |  |  |  |  |  | X |  | X |  | X | X |
| Sorondo 2016 |  |  |  |  |  |  |  |  | X | X |  |
| Johansen 2019 |  |  |  | X |  |  |  |  |  |  |  |
| Grossman 2018 |  |  |  |  |  |  |  |  |  |  |  |
| Graetz 2020 |  |  |  |  |  |  | X |  | X | X |  |
| Wang 2020 | X | X | X | X |  |  |  |  |  | X |  |
| Bryan 2020 |  |  |  |  |  |  |  |  |  | X |  |
| Nicolas 2019 |  |  |  |  |  |  |  |  | X |  |  |
| Graham 2020 |  |  |  |  |  |  |  |  |  | X | X |
| Moll 2019 |  |  |  |  |  |  |  |  |  | X |  |
| Zanaboni 2020 | X | X |  | X | X |  |  |  |  | X |  |
| Zhong 2020 |  |  |  |  |  |  |  |  |  | X |  |
| Garry 2020 |  |  |  |  |  |  |  |  |  | X |  |
| Szilagyi 2020 | X | X |  |  |  |  |  |  |  | X |  |
| Fiks 2015 |  |  |  |  | X |  |  |  |  | X | X |
| Ronda 2015 |  |  |  |  |  |  |  |  |  | X | X |
| Crotty 2015 |  |  |  |  |  |  | X | X |  | X |  |
| Shah 2015 |  |  |  |  |  |  |  |  |  | X | X |
| Saberi 2015 |  |  |  |  |  |  | X | X |  | X |  |
| Wright 2015 |  |  |  |  |  |  |  | X |  | X | X |
| KummerowBroman 2015 |  |  |  |  |  |  | X | X |  | X |  |
| Jhamb 2015 |  |  |  |  |  |  |  |  |  | X | X |
| Riippa 2015 |  |  |  |  |  |  |  |  | X |  |  |
| Haun 2015 |  |  |  |  |  |  |  |  |  | X | X |
| Raghu 2015 |  |  |  |  |  |  |  |  |  | X | X |
| Giardina 2015 |  |  |  |  |  |  |  |  |  | X | X |
| Mendel 2017 |  | X |  |  |  |  |  |  |  |  |  |
| Sieck 2017 |  |  |  |  |  |  |  |  |  | X | X |
| Vydra 2015 |  |  |  |  |  |  |  |  |  |  |  |
| Reed 2015 |  |  |  |  |  |  |  |  |  | X | X |
| Forster 2015 |  |  |  |  |  |  |  |  |  | X | X |
| Wildenbos 2018 |  |  |  |  |  |  |  |  |  | X | X |
| Esch 2016 |  |  |  |  |  |  |  |  |  | X | X |
| Mafi 2016 | X |  |  |  |  |  |  |  |  | X | X |
| Pillemer 2016 |  |  |  | X | X | X | X | X |  | X | X |
| Grunloh 2016 |  |  |  |  |  |  |  |  |  | X |  |
| Shimada 2016 |  |  |  |  |  |  |  |  | X | X |  |
| Winget 2016 |  |  |  |  |  |  |  |  |  | X |  |
| Manard 2016 |  |  |  |  |  |  |  |  | X | X |  |
| Petullo 2016 |  |  |  |  |  |  |  | X | X | X |  |
| Griffin 2016 |  |  |  |  |  |  | X |  | X | X | X |
| Devkota 2016 |  |  |  |  |  |  |  |  | X | X |  |
| Lyles 2016 |  |  |  |  |  |  | X | X | X |  |  |
| Millman 2016 |  |  |  |  |  |  | X | X | X |  |  |
| Leveille 2016 |  |  |  |  |  |  |  |  |  |  |  |
| Reicher 2016 |  |  |  |  |  |  |  |  |  |  |  |
| Dexter 2016 |  |  |  |  |  |  |  |  |  |  |  |
| Rief 2017 |  |  |  |  |  |  |  |  |  | X | X |
| Hanna 2017 |  |  |  |  |  |  |  |  |  | X | X |
| Federman 2017 |  |  |  | X | X | X |  | X |  | X | X |
| Wolff 2017 |  |  |  | X | X |  |  |  |  | X | X |
| Lieu 2019 |  |  |  |  |  |  |  |  |  | X |  |
| Peremislov 2016 |  |  |  |  |  |  | X | X |  | X |  |
| Akerstedt 2018 |  |  |  |  |  |  |  |  |  | X |  |
| Zhou 2015 |  |  |  |  |  |  |  |  |  | X |  |
| Crouch 2015 |  |  |  |  |  |  |  |  |  | X | X |

| **Study** | **Productivity** | | | **Usage** | | | **User Satisfaction** | | |
| --- | --- | --- | --- | --- | --- | --- | --- | --- | --- |
|  | Efficiency | Care coordination | Net cost | Use Behavior/Pattern | Self-Reported Use | Intention to Use | Competency | User Satisfaction | Ease of Use |
| Shaw 2017 |  |  |  | X |  | X |  |  | X |
| Gerard 2017 |  |  |  | X | X | X | X | X | X |
| Giardina 2018 |  |  |  |  | X |  |  | X | X |
| Dumitrascu 2018 |  |  | X | X |  | X |  |  |  |
| Grunloh 2018 | X | X |  | X |  |  | X | X | X |
| Kayastha 2018 |  |  |  | X | X | X | X | X | X |
| Bush 2017 |  |  |  |  |  |  |  |  |  |
| King 2017 | X |  |  |  | X | X | X | X | X |
| Denneson 2018 |  |  |  | X | X |  | X | X | X |
| Chimowitz 2018 |  |  |  | X | X |  |  | X | X |
| Cajander 2018 |  |  |  |  |  |  |  |  |  |
| Zhong 2018 | X | X | X | X |  |  |  |  |  |
| Moll 2018 |  |  |  | X | X | X |  | X |  |
| Plate 2019 |  |  | X | X |  |  |  |  |  |
| Shah 2019 | X |  | X |  |  |  |  |  |  |
| Robinson 2019 |  |  |  | X | X |  |  | X | X |
| Fossa 2018 |  |  |  |  | X | X |  |  |  |
| Reed 2019 |  |  |  | X | X | X | X | X | X |
| Cutrona 2018 | X |  |  | X |  |  |  |  |  |
| Walker 2019 |  |  |  | X | X | X | X | X | X |
| Foster 2019 |  |  | X |  |  |  |  |  |  |
| Dalal 2019 | X | X |  |  |  |  |  |  |  |
| Sorondo 2016 |  |  | X | X |  |  |  |  |  |
| Johansen 2019 |  |  |  |  |  |  |  |  |  |
| Grossman 2018 |  |  |  |  |  |  |  |  |  |
| Graetz 2020 |  |  |  | X |  |  |  |  |  |
| Wang 2020 | X |  |  | X | X | X | X | X | X |
| Bryan 2020 | X |  | X |  |  |  |  |  |  |
| Nicolas 2019 |  |  | X |  |  |  |  |  |  |
| Graham 2020 |  |  | X |  | X |  |  | X |  |
| Moll 2019 |  |  |  | X |  |  |  |  |  |
| Zanaboni 2020 |  |  |  | X | X | X | X | X | X |
| Zhong 2020 |  |  | X | X |  |  |  | X |  |
| Garry 2020 |  |  |  |  | X |  | X | X | X |
| Szilagyi 2020 |  |  |  | X |  |  |  |  |  |
| Fiks 2015 |  |  |  | X | X | X | X | X | X |
| Ronda 2015 |  |  |  | X | X | X | X | X | X |
| Crotty 2015 | X | X |  | X |  |  |  |  |  |
| Shah 2015 |  |  |  | X | X | X | X | X | X |
| Saberi 2015 |  |  |  |  |  |  |  |  |  |
| Wright 2015 |  |  |  | X |  |  |  |  |  |
| KummerowBroman 2015 |  | X |  |  |  |  |  | X |  |
| Jhamb 2015 |  |  |  | X |  |  |  |  |  |
| Riippa 2015 |  |  | X | X |  |  |  |  |  |
| Haun 2015 |  |  |  | X | X | X | X | X | X |
| Raghu 2015 |  |  |  | X | X | X |  |  |  |
| Giardina 2015 |  |  |  | X | X | X | X | X | X |
| Mendel 2017 | X |  | X |  | X | X |  |  |  |
| Sieck 2017 | X | X |  | X | X | X | X | X | X |
| Vydra 2015 | X | X |  |  |  |  |  |  |  |
| Reed 2015 |  |  |  | X | X |  | X | X |  |
| Forster 2015 |  |  |  | X | X | X | X | X | X |
| Wildenbos 2018 |  |  |  | X | X | X | X | X | X |
| Esch 2016 |  |  |  | X | X | X | X | X |  |
| Mafi 2016 |  |  |  | X |  |  |  |  |  |
| Pillemer 2016 | X |  | X | X |  |  | X | X |  |
| Grunloh 2016 | X |  |  |  |  |  |  |  |  |
| Shimada 2016 |  |  |  |  |  |  |  |  |  |
| Winget 2016 | X |  |  |  |  |  |  |  |  |
| Manard 2016 |  |  |  | X |  |  |  |  |  |
| Petullo 2016 |  |  | X |  |  |  |  |  |  |
| Griffin 2016 |  |  | X | X |  |  |  |  |  |
| Devkota 2016 |  |  |  | X |  |  |  |  |  |
| Lyles 2016 |  |  |  | X |  |  |  |  |  |
| Millman 2016 |  |  |  |  |  |  |  |  |  |
| Leveille 2016 | X |  |  |  |  |  |  |  |  |
| Reicher 2016 |  | X |  | X |  |  |  | X |  |
| Dexter 2016 | X |  | X |  |  |  |  |  |  |
| Rief 2017 |  |  |  | X | X |  | X | X | X |
| Hanna 2017 |  |  |  | X | X | X | X | X | X |
| Federman 2017 | X | X |  | X | X | X | X | X | X |
| Wolff 2017 |  |  |  | X |  |  |  | X | X |
| Lieu 2019 | X |  |  |  |  |  |  |  |  |
| Peremislov 2016 |  |  |  | X |  |  |  |  |  |
| Akerstedt 2018 |  |  |  |  |  |  |  |  |  |
| Zhou 2015 | X |  | X |  |  |  |  |  |  |
| Crouch 2015 |  |  |  |  | X |  |  | X |  |

| **Study** | **Patient** | | | **Provider** | | **Change/ Improvement** | **Implementation** | |
| --- | --- | --- | --- | --- | --- | --- | --- | --- |
|  | Knowledge, attitude, perception, decision confidence, compliance | Overall satisfaction | Knowledge acquisition, relationship | Attitude, perceptions, autonomy, experience and performance | Workflow | Data quality improvement, reduced loss/paper and transcription errors | | Barriers, training, organizational support, time-to-evaluation, lessons, success factors, project management, leadership, costs |
| Shaw 2017 |  |  |  | X | X |  | | X |
| Gerard 2017 | X | X | X |  |  |  | |  |
| Giardina 2018 |  | X | X |  |  |  | |  |
| Dumitrascu 2018 |  |  |  |  |  |  | |  |
| Grunloh 2018 | X |  | X | X | X |  | | X |
| Kayastha 2018 | X | X | X |  |  |  | |  |
| Bush 2017 |  |  |  | X | X |  | |  |
| King 2017 | X | X | X | X | X |  | |  |
| Denneson 2018 | X | X | X |  |  |  | |  |
| Chimowitz 2018 | X | X | X |  |  |  | |  |
| Cajander 2018 |  |  |  | X | X |  | |  |
| Zhong 2018 |  |  |  |  |  |  | |  |
| Moll 2018 | X | X |  |  |  |  | |  |
| Plate 2019 |  |  |  |  | X |  | |  |
| Shah 2019 |  |  |  |  |  |  | |  |
| Robinson 2019 | X | X | X |  |  |  | |  |
| Fossa 2018 | X |  |  |  |  |  | |  |
| Reed 2019 | X | X | X | X |  |  | |  |
| Cutrona 2018 |  |  |  |  | X |  | |  |
| Walker 2019 |  |  |  |  |  |  | |  |
| Foster 2019 |  |  |  |  |  |  | |  |
| Dalal 2019 |  |  |  |  |  |  | |  |
| Sorondo 2016 |  |  |  |  |  |  | |  |
| Johansen 2019 |  |  |  | X | X |  | |  |
| Grossman 2018 |  |  |  | X | X |  | |  |
| Graetz 2020 |  |  |  |  |  |  | |  |
| Wang 2020 | X | X |  | X | X |  | | X |
| Bryan 2020 |  |  |  |  | X |  | |  |
| Nicolas 2019 |  |  |  |  |  |  | |  |
| Graham 2020 |  | X |  |  |  |  | |  |
| Moll 2019 |  |  |  | X | X |  | |  |
| Zanaboni 2020 | X | X | X |  |  |  | |  |
| Zhong 2020 |  |  |  |  |  |  | |  |
| Garry 2020 | X | X | X |  |  |  | |  |
| Szilagyi 2020 |  |  |  |  |  |  | |  |
| Fiks 2015 | X | X | X |  |  |  | |  |
| Ronda 2015 | X | X | X |  |  |  | |  |
| Crotty 2015 |  |  |  |  | X |  | |  |
| Shah 2015 | X | X | X |  |  |  | |  |
| Saberi 2015 |  |  |  |  |  |  | |  |
| Wright 2015 |  |  |  |  |  |  | |  |
| KummerowBroman 2015 |  | X |  | X | X |  | |  |
| Jhamb 2015 |  |  |  |  |  |  | |  |
| Riippa 2015 |  |  |  |  |  |  | |  |
| Haun 2015 | X | X | X |  |  |  | |  |
| Raghu 2015 |  |  |  |  |  |  | |  |
| Giardina 2015 | X | X | X |  |  |  | |  |
| Mendel 2017 |  | X |  |  |  |  | | X |
| Sieck 2017 | X | X | X |  |  |  | |  |
| Vydra 2015 |  |  |  | X | X |  | |  |
| Reed 2015 |  | X | X |  |  |  | |  |
| Forster 2015 | X | X | X |  |  |  | |  |
| Wildenbos 2018 | X | X | X | X |  |  | |  |
| Esch 2016 | X | X | X |  |  |  | |  |
| Mafi 2016 |  |  |  |  |  |  | |  |
| Pillemer 2016 |  | X | X | X | X |  | |  |
| Grunloh 2016 |  |  |  | X | X |  | |  |
| Shimada 2016 |  |  |  |  |  |  | |  |
| Winget 2016 |  |  |  | X | X |  | |  |
| Manard 2016 | X |  | X |  |  |  | |  |
| Petullo 2016 |  |  |  |  |  |  | |  |
| Griffin 2016 |  |  |  |  |  |  | |  |
| Devkota 2016 | X |  |  |  |  |  | |  |
| Lyles 2016 |  |  |  |  |  |  | |  |
| Millman 2016 |  |  |  |  |  |  | |  |
| Leveille 2016 |  |  |  |  |  |  | |  |
| Reicher 2016 |  |  |  |  |  | X | |  |
| Dexter 2016 |  |  |  |  |  | X | |  |
| Rief 2017 | X | X | X |  |  |  | |  |
| Hanna 2017 | X | X | X |  |  |  | |  |
| Federman 2017 | X | X | X | X | X |  | |  |
| Wolff 2017 | X | X |  |  |  |  | |  |
| Lieu 2019 |  |  |  | X | X |  | |  |
| Peremislov 2016 |  |  |  |  | X |  | |  |
| Akerstedt 2018 |  |  |  | X | X |  | |  |
| Zhou 2015 |  |  |  |  |  |  | |  |
| Crouch 2015 | X | X | X |  |  |  | |  |
